# Transmission Bottleneck Size Estimation from De Novo Viral Genetic Variation

**DOI:** 10.1093/molbev/msad286

**Published:** 2023-12-30

**Authors:** Yike Teresa Shi, Jeremy D Harris, Michael A Martin, Katia Koelle

**Affiliations:** Department of Biology, Emory University, Atlanta, GA, USA; Department of Biology, Emory University, Atlanta, GA, USA; Department of Biology, Emory University, Atlanta, GA, USA; Graduate Program in Population Biology, Ecology, and Evolution, Emory University, Atlanta, GA, USA; Department of Biology, Emory University, Atlanta, GA, USA; Emory Center of Excellence for Influenza Research and Response (CEIRR), Atlanta, GA, USA

**Keywords:** transmission bottleneck, viral evolution, transmission pairs, SARS-CoV-2, influenza A virus

## Abstract

Sequencing of viral infections has become increasingly common over the last decade. Deep sequencing data in particular have proven useful in characterizing the roles that genetic drift and natural selection play in shaping within-host viral populations. They have also been used to estimate transmission bottleneck sizes from identified donor–recipient pairs. These bottleneck sizes quantify the number of viral particles that establish genetic lineages in the recipient host and are important to estimate due to their impact on viral evolution. Current approaches for estimating bottleneck sizes exclusively consider the subset of viral sites that are observed as polymorphic in the donor individual. However, these approaches have the potential to substantially underestimate true transmission bottleneck sizes. Here, we present a new statistical approach for instead estimating bottleneck sizes using patterns of viral genetic variation that arise de novo within a recipient individual. Specifically, our approach makes use of the number of clonal viral variants observed in a transmission pair, defined as the number of viral sites that are monomorphic in both the donor and the recipient but carry different alleles. We first test our approach on a simulated dataset and then apply it to both influenza A virus sequence data and SARS-CoV-2 sequence data from identified transmission pairs. Our results confirm the existence of extremely tight transmission bottlenecks for these 2 respiratory viruses.

## Introduction

In viral infections, transmission bottleneck sizes are defined as the number of viral particles transmitted from a donor to a recipient host that successfully establish genetic lineages within the recipient. Quantifying the magnitude of these bottlenecks is important for understanding the ecological and evolutionary dynamics of viruses at multiple scales, as these bottlenecks bridge processes occurring at within-host and between-host levels (Zwart and Elena 2015; McCrone and Lauring 2018). At the level of the population, tight transmission bottlenecks can act to slow down the rate of viral adaptation, as beneficial mutations that arise within a donor host can be lost during transmission to a recipient host (Abel et al. 2015; Zaraket et al. 2015; Zwart and Elena 2015; Geoghegan et al. 2016). However, they may also be advantageous to a viral population, for example by enabling its path through a rugged fitness landscape and by purging cheaters from its population (Zwart and Elena 2015). At the within-host level, tight transmission bottlenecks lead to lower levels of viral genetic diversity in recipient hosts and genetic drift playing an important role in shaping the viral population during the early stages of a recipient’s infection (Gutiérrez et al. 2012; Abel et al. 2015; Zwart and Elena 2015; McCrone and Lauring 2018; McCrone et al. 2018). Finally, quantifying transmission bottleneck sizes is important for more applied reasons: having estimates of the bottleneck size may help determine whether it is possible to reconstruct who-infected-whom in an outbreak setting and will determine which inference methods might be the most suitable to use in a specific application (Hall et al. 2016; Campbell et al. 2018; Duault et al. 2022).

Several statistical methods have recently been developed to estimate transmission bottleneck sizes from viral deep-sequencing data (Emmett et al. 2015; Zwart and Elena 2015; Sobel Leonard et al. 2017b; Ghafari et al. 2020). All of these approaches rely on patterns of shared genetic variation by first characterizing the genetic variation that is present in both the donor and the recipient of an identified transmission pair. They then restrict their analyses to the subset of sites that are polymorphic in the donor. One approach (the presence/absence method) estimates bottleneck sizes by asking which of the variants identified in the donor are also detected in the recipient and which are not. A second approach (the binomial sampling method) instead makes use of variant frequencies quantified in the recipient, rather than just their presence or absence. However, it assumes that the observed differences in variant frequencies between a donor and a recipient arise from the process of viral sampling alone (Emmett et al. 2015; Poon et al. 2016). A third approach (the betabinomial sampling method) similarly makes use of variant frequencies from the recipient but additionally accounts for deviations between donor and recipient variant frequencies that arise from demographic noise during the early period of exponential viral growth in the recipient (Sobel Leonard et al. 2017b). Finally, a haplotype-based approach to transmission bottleneck size estimation has been developed (Ghafari et al. 2020); it extends the betabinomial sampling method to account for genetic linkage between loci.

Applications of these inference methods to viral sequence data have indicated that transmission bottlenecks are tight for many viral pathogens. Several studies have estimated bottleneck sizes of 1–3 viral particles for plant viruses (Moury et al. 2007; Betancourt et al. 2008; Sacristán et al. 2011). Tight transmission bottlenecks of 1–5 viral particles have also been estimated for human viruses, including influenza viruses (McCrone et al. 2018; Valesano et al. 2020), HIV-1 (Keele et al. 2008), and most recently SARS-CoV-2 (Braun et al. 2021a, 2021b; Lythgoe et al. 2021; Martin and Koelle 2021; Nicholson et al. 2021; Wang et al. 2021; Li et al. 2022; Bendall et al. 2023). When bottlenecks are tight, as in these cases, there is little genetic diversity that is transferred from a donor to a recipient. For acute infections, with little time to accrue new mutations, this often times leads to overall low levels of viral diversity in infected hosts. When there is no viral genetic diversity observed in a donor sample, estimation of transmission bottleneck size is not possible for that transmission pair. Studies that estimate bottleneck sizes (such as the ones cited above) therefore often rely on combining data from across a large number of transmission pairs to quantify an average bottleneck size. Within experimental settings, barcoded viruses can be used to increase host genetic diversity and thereby to improve resolution of transmission bottleneck sizes (Varble et al. 2014; Amato et al. 2022). However, natural settings do not afford us with this possibility.

Three issues need to be considered when interpreting bottleneck size estimates derived from inference methods that rely on patterns of shared genetic variation. One issue is that the time of the infectious contact is not known in many cases, and the donor is unlikely to be sampled exactly at the point of transmission. Longitudinal studies of acute infections have indicated that variant frequencies can change rapidly over the course of infection, with many variants that are observed on one day not being observed on an adjacent day (McCrone et al. 2018; Popa et al. 2020; Valesano et al. 2020). These rapid variant frequency changes in the donor will act to considerably depress inferred bottleneck size estimates, as the assumed allele frequencies at the time of transmission will deviate from the true ones. A second issue is that rapid variant frequency changes in the recipient will similarly act to depress inferred bottleneck size estimates, as transmitted genetic variation could be lost in the recipient even when viral titers are high. The extent of shared genetic variation between a donor and a recipient may therefore be more indicative of the extent of viral genetic drift within individual infections than the size of the transmission bottleneck. A third issue is that existing methods all assume that viral particles that initiate infection in the recipient are randomly sampled from the donor. However, it could be the case that genetically similar virions are aggregated and transmit together, as would be the case with collective infectious units (Sanjuán 2017). If this is the case, one would again erroneously infer bottleneck sizes to be tight when they might in fact be loose.

Here, we develop an approach for estimating transmission bottleneck sizes that instead makes use of de novo genetic variation that is observed in a recipient. Similar to some existing approaches, it assumes that all observed genetic variation is neutral and that the viral population in the recipient host undergoes stochastic exponential growth. It differs from existing approaches, however, in that it uses a different subset of sites for inference, namely sites that are monomorphic in both the donor and recipient but carry different alleles. Consideration of these sites, rather than sites that are polymorphic in the donor, circumvents the 3 issues described above. To introduce our approach, we first describe the stochastic model that we assume underlies the process of viral population expansion in a recipient. We then describe the inference framework and test our approach on simulated data, showing that it accurately recovers transmission bottleneck sizes. Finally, we apply our approach to data from influenza A virus (IAV) and SARS-CoV-2 transmission pairs, confirming previous findings of tight transmission bottlenecks for these respiratory viruses using an approach that is not prone to underestimating this quantity.

## Materials and Methods

### The Stochastic Within-Host Model

We model the dynamics of the viral population within a recipient using a multitype branching process model. The types in this model correspond to different viral genotypes. Because we assume that all mutations are neutral, each type has the same overall offspring distribution. More specifically, we assume a geometric offspring distribution, consistent with the offspring distribution under a stochastic birth–death model. The geometric distribution is parameterized with a success probability of $p_{\mathrm{geom}}$, where $p_{\mathrm{geom}}=1/(R_{0}+1)$ and $R_{0}$ is the within-host basic reproduction number. As such, the expected number of offspring a given viral particle leaves is given by $R_{0}$. The number of mutations that occur during the production of a viral offspring is assumed to be Poisson-distributed with mean *μ*. When one or more mutations occur during the production of an offspring, the resultant offspring becomes a new type. As such, we assume infinite sites. In addition to carrying any new mutations, offspring inherit the mutations of their parent. Because we model the virus population as asexually reproducing, genetic linkage across the virus genome is assumed to be complete. Because we are interested in characterizing transmission dynamics between infections, we consider only the supercritical case corresponding to a within-host basic reproduction number of $R_{0}>1$.

The virus population starts with an initial population size of *N* viral particles, which stem from the donor’s virus population. *N* is related, but not equivalent, to the transmission bottleneck size $N_{b}$. This is because $N_{b}$ quantifies the number of viral particles that succeed in establishing a genetic lineage in the recipient and some of the *N* initial viral particles may have lineages that do not successfully establish but instead go stochastically extinct. As such $N_{b}\leq N$. All *N* initial viral particles harbor 0 de novo mutations, where we define de novo mutations as mutations that occurred during viral replication in the recipient. These *N* initial viral particles could in principle be genetically distinct from one another. Any genetic variation that is present in these particles stems from the donor. Hereafter, we refer to viral particles without de novo mutations (including these *N* initial viral particles) as wild-type particles, while remaining cognizant that these could differ from one another genetically. We further define a wild-type lineage as a genetic lineage that starts from an initial wild-type particle and includes the subset of offspring that are wild-type. Finally, we define a mutant lineage as a lineage that starts from a viral particle that is an immediate descendant of a wild-type particle and carries one or more mutations relative to that wild-type parent.

**Fig. 1.**
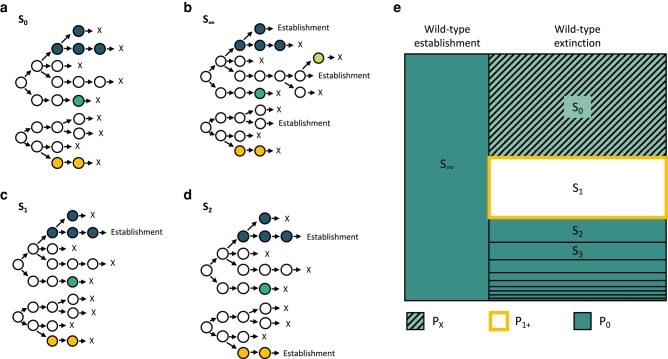
Possible dynamic outcomes in the recipient. a) The viral population in the recipient may go stochastically extinct, leading to no infection in the recipient. b) One or more wild-type lineages may successfully establish. c) No wild-type lineages establish but a single mutant lineage successfully establishes. d) No wild-type lineages establish but 2 or more mutant lineages successfully establish. Here, we show a scenario of 2 mutant lineages successfully establishing. Outcomes (b–d) result in successful infection of the recipient. Wild-type particles are shown in white. Mutant lineages are shown in different colors. In (a–d), N=2 initial viral particles and $S_{l}$ denotes the number of mutant lineages *l* that successfully establish under each scenario. e) Summary of possible dynamic outcomes, with $S_{l}$ again denoting the number of mutant lineages *l* that establish. Outcomes are color-coded by the number of clonal variants *k* that would be observed under the outcome. The portion of the outcome space labeled $P_{X}$ denotes the probability that the viral population in the recipient goes extinct. The portion of the outcome space labeled $P_{0}$ denotes the probability that *k* = 0 clonal variants establish in the recipient’s viral population. The portion of the outcome space labeled $P_{1^{+}}$ denotes the probability that at least 1 clonal variant establishes in the recipient’s viral population.

We can lay out all of the possible dynamic outcomes of this branching process model. The first possible outcome is that the virus population in the recipient goes stochastically extinct (Fig. 1a). This would result in the recipient remaining uninfected and (necessarily, but trivially) 0 mutant lineages successfully establishing in the recipient. The second possible outcome is that at least one of the *N* initial viral particles seeds a wild-type lineage that successfully establishes (Fig. 1b). In this case, there will theoretically be an infinite number of mutant lineages that will successfully establish. This is because under a supercritical branching process the wild-type viral population will ultimately grow geometrically at a per generation rate of $R_{0}\;e^{-\mu }$, and each of the wild-type viral particles in this ever-growing population may give rise to a mutant lineage that will also establish in the viral population. In reality, viral population sizes will expand and then decline in an acute infection, such that there will be many, but not an infinite number of mutant viral lineages that establish initially, only to die out toward later stages of an infection. The third possible outcome is that no wild-type viral lineages establish but a single mutant lineage, seeded by a wild-type viral particle, establishes (Fig. 1c). Finally, the fourth possible outcome is that no wild-type viral lineages establish but 2 or more mutant lineages establish (Fig. 1d). In the case of a successful infection (Fig. 1b–d), the overall viral population will grow geometrically at rate $R_{0}$ once the population has reached a large size. Again, in reality, viral population sizes in an acute infection will increase and then decrease. Successful sequencing of a viral sample from a recipient, however, will occur when viral titers are still relatively high.

For a given outcome, we can quantify the number of variants that arose and fixed in the viral population of the recipient. We refer to these variants as *clonal* variants. In the case of the viral population going extinct (the first outcome; Fig. 1a), the infection in the recipient did not establish and we will not have observed this outcome in a transmission pair. We refer to the probability of this outcome as $P_{X}$. In the case of one or more of the wild-type viral lineages establishing (the second outcome; Fig. 1b), the number of clonal variants will be 0, because none of the mutations that arose in any of the mutant lineages will fix. In the case of no wild-type viral lineages establishing but 2 or more mutant lineages successfully establishing (the fourth outcome; Fig. 1d), the number of clonal variants will similarly be 0, because none of the mutations that arose in any of the mutant lineages will fix under an infinite sites assumption. Finally, in the case of no wild-type viral lineages establishing but exactly one mutant lineage successfully establishing (the third outcome; Fig. 1c), the number of clonal variants will be at least one. It will be exactly one if only a single mutation occurred during the generation of the mutant lineage and no additional clonal variants arose in this mutant lineage. It will be greater than one if more than one mutation occurred during the generation of the mutant lineage and/or if additional mutations occurred in this mutant lineage that ultimately fixed. Figure 1e graphically summarizes all of these possible dynamic outcomes.

### Derivation of the Probability Distribution for the Number of Clonal Variants

The multitype branching process model, resulting in the different possible outcomes shown in Fig. 1, contains 3 parameters: the initial wild-type viral population size *N*, the within-host basic reproduction number $R_{0}$, and the per genome, per infection cycle mutation rate *μ*. Here, we are specifically interested in estimating the initial viral population size *N*. Estimates of *N* will be used to calculate the transmission bottleneck size $N_{b}$. To estimate *N*, we need to ask: for a given recipient harboring *k* clonal variants, what is the likelihood that the initial viral population size was N=1,2,3,…? These likelihoods can be calculated if we can calculate the probability distribution for a recipient harboring k=0,1,2,… clonal variants, for given values of *N*, $R_{0}$, and *μ*. In the Supplementary material, we derive the expression for this probability distribution based on the different possible dynamic outcomes shown in Fig. 1e, with associated supplementary fig. S1, Supplementary Material online graphically depicting the steps involved in this derivation.

**Fig. 2.**
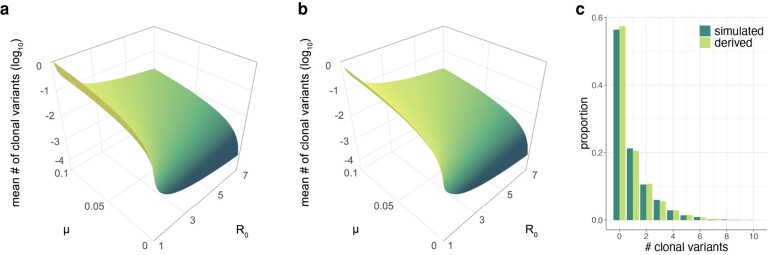
Confirmation of our analytical results. a) The expected number of clonal mutations when N=1, as derived by Bozic et al. (2016) using a birth–death model. Mean numbers of clonal mutations are shown across a range of within-host $R_{0}$ and *μ* parameter values. b) The mean number of clonal mutations, as calculated from our analytical expressions, parameterized with N=1. c) Histogram showing the proportion of simulations that resulted in k=0,1,2,… clonal variants (dark green), alongside our analytical predictions (light green). Simulated proportions were calculated using 4,000 stochastic simulations that resulted in successful infection. Simulations and analytical results shown in panel c were parameterized with N=2, $R_{0}=1.2$, and μ=0.2.

We can confirm the accuracy of our analytical results in 2 ways. First, previous work by Bozic et al. (2016), in the context of cancer cell dynamics, derived an equation for the number of clonal variants one would expect in a population undergoing birth–death dynamics, given an initial population size of N=1. This expected number is given by δu/(1−δ), where their parameter *δ* corresponds to $1/R_{0}$ and their mutation parameter *u* corresponds to our *μ*, under the assumption that *μ* is small (≪1). Figure 2a shows the expected number of clonal variants across a range of within-host $R_{0}$ and across a range of mutation rates *μ*, as calculated from their equation. In Fig. 2b, we plot the expected number of clonal variants as given by our analytical results under the assumption of N=1. The quantitative similarity of the plots shown in Fig. 2a and b demonstrates the accuracy of our clonal variant derivation. In the Supplementary material, we further show how we can derive their equation using our analytical expressions, under the assumption of a low mutation rate.

The second way we can check our analytical results is through extensive numerical simulation of the branching process model. For a given simulation, we can determine whether the viral population went stochastically extinct or whether infection was successful. For those simulations establishing successful infection, we can determine the number of clonal variants that evolved. To check our clonal variant derivation, we plot in Fig. 2c the fraction of simulations that resulted in k=0,1,2,… clonal variants from 4,000 simulations that were each parameterized with an initial viral population size of N=2, a within-host basic reproduction number of $R_{0}=1.2$, and a per genome per infection cycle mutation rate of μ=0.2. Alongside this empirical distribution, we plot the analytically derived clonal variant probabilities under this parameterization. The quantitative similarity of these distributions demonstrates the accuracy of our analytical derivations.

## Results

### Application to Simulated Data

**Fig. 3.**
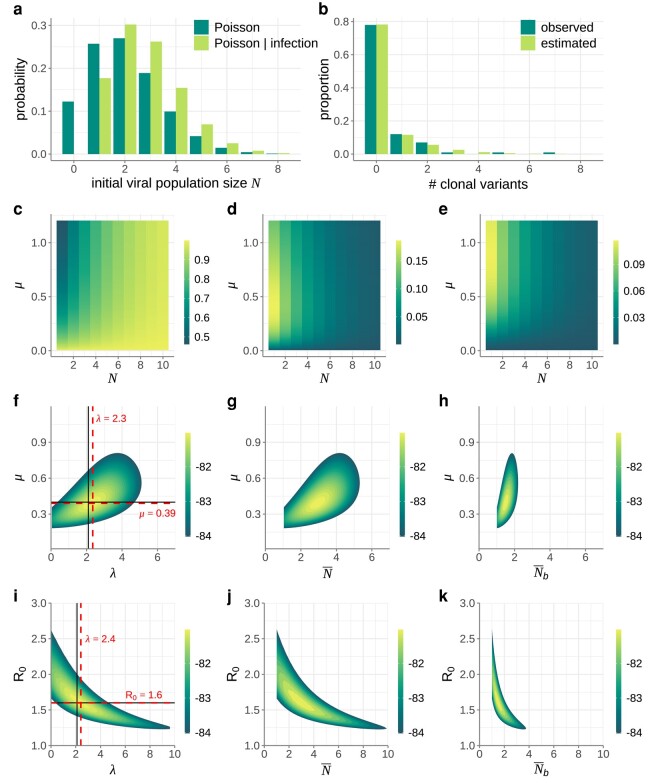
Application of our inference method to a mock dataset of 100 transmission pairs. a) Poisson probability distribution showing the distribution of initial viral population sizes *N* that seed potential recipient infections (dark green bars). Here, the mean of this Poisson distribution is λ=2.1. The probability distribution of the initial viral population size being *N*, conditional on successful infection, is also shown (light green bars). b) Proportion of simulated infections that resulted in k=0,1,2,… clonal variants (dark green bars). Of the 100 simulated infections, 78 recipients had no clonal variants, 12 recipients had 1 clonal variant, 7 recipients had 2 clonal variants, 1 recipient had 3 clonal variants, 1 recipient had 5 clonal variants and 1 recipient had 7 clonal variants. Alongside the simulated (observed) data, we show the proportion of infections with k=0,1,2,… clonal variants that we estimated using maximum likelihood values λ=2.3 and μ=0.39 (light green bars). c) Probabilities of observing k=0 clonal variants across a range of *N* and *μ* values. d) Probabilities of observing k=1 clonal variant across a range of *N* and *μ* values. e) Probabilities of observing k=2 clonal variants across a range of *N* and *μ* values. f) Log-likelihood plot, showing the log(probability) of observing the mock dataset given parameters *λ* and *μ*. Black lines show the true values of *λ* and *μ*. Dashed red lines show the maximum likelihood values of *λ* and *μ*. g) Log-likelihood plot, as in panel f, with the results plotted as a function of $\bar{N}$ and *μ*. h) Log-likelihood plot, as in panel f, with the results plotted as a function of $\bar{N_{b}}$ and *μ*. In f–h), $R_{0}$ was set to its true value of 1.6. i) Log-likelihood plot, showing the log(probability) of observing the mock dataset given parameters *λ* and $R_{0}$. Black lines show the true values of *λ* and $R_{0}$. Dashed red lines show the maximum likelihood values of *λ* and $R_{0}$. j) Log-likelihood plot, as in panel i, with the results plotted as a function of $\bar{N}$ and $R_{0}$. k) Log-likelihood plot, as in panel i, with the results plotted as a function of $\bar{N_{b}}$ and $R_{0}$. In i–k), the mutation rate was set to its true value of 0.4 mutations per genome per infection cycle. In f–k), log-likelihood values are shown only for the parameter combinations that fall within the 95% confidence region.

Before applying our statistical method to sequence data from empirical transmission pair studies, we first applied our approach to simulated (mock) data. To this end, we forward simulated the branching process model until we obtained 100 successful recipient infections. Forward simulations were all performed with a within-host basic reproduction number of $R_{0}=1.6$ and a per genome, per infection cycle mutation rate of μ=0.4. Instead of assuming that the initial viral population size *N* was the same across all recipients, we assumed that the initial number of viral particles was Poisson-distributed with mean λ=2.1 (Fig. 3a). (Simulations with a higher *N* had a lower chance of going stochastically extinct so higher *N* simulations were overrepresented in the mock dataset, which we account for, as described in greater detail below.)

For each of these 100 simulated successful infections, we calculated the number of clonal variants present in the recipient viral population once large. Figure 3b shows the proportion of these 100 simulations that resulted in k=0,1,2,… clonal variants. We then set $R_{0}$ to its true value of 1.6 and attempted to jointly estimate *λ* and *μ* from this observed mock dataset. To do this, we first calculated across combinations of *N* and *μ* the probability of observing k=0 clonal variants (Fig. 3c), k=1 clonal variant (Fig. 3d), k=2 clonal variants (Fig. 3e), k=3 clonal variants (not shown), k=5 clonal variants (not shown), and k=7 clonal variants (not shown). We did not perform the calculation for other values of *k* because there were no simulated infections that resulted in these other numbers of clonal variants.

From the mock dataset shown in Fig. 3b, our goal was then to estimate *λ* and *μ* given knowledge of the within-host basic reproduction number $R_{0}$. To do this, we first adjusted the Poisson distribution shown in Fig. 3a (dark green bars) to reflect the distribution of initial viral population sizes we would expect across *successful* infections (Fig. 3a, light green bars). This adjustment involved multiplying the Poisson probability masses by the *N*-specific probabilities of successful establishment ($1-(1/R_{0}{)}^{N}$) and renormalizing. For a given transmission pair, the probability that a recipient’s viral population harbors *k* clonal variants is then given by


(1) $$\text{Prob}(k|\lambda ,\mu ,R_{0})=\sum_{N=0}^{\infty }p_{N}(\lambda )\rho_{k}(N,\mu ,R_{0}),$$

where $p_{N}(\lambda )$ is the probability that *N* viral particles started off a successful viral infection under an assumed Poisson distribution with mean *λ* (Fig. 3a, light green bars) and $\rho_{k}(N,\mu ,R_{0})$ is the probability that the recipient’s viral population harbors *k* clonal variants. We can calculate this probability for each of the 100 transmission pairs in our mock dataset, and then calculate the overall log-likelihood of observing the data shown in Fig. 3b (dark green bars) by summing the log of these probabilities. In Fig. 3f, we plot this log-likelihood surface over a broad range of *λ* values and *μ* values, while setting the within-host basic reproduction number $R_{0}$ to its true value of 1.6. The estimated values of *μ* and *λ* are very close to their true values and the 95% confidence intervals include the true value. As such, these results indicate that our inference approach performs well on this simulated dataset of 100 transmission pairs.

In addition to plotting the log-likelihood landscape as a function of *λ* and *μ*, we can plot the same results as a function of the mean realized initial viral population size $\bar{N}$ and *μ* (Fig. 3g). The mean realized initial viral population size quantifies the mean number of initial viral particles across successful infections and as such corresponds to the mean of the adjusted Poisson distribution shown in Fig. 3a (light green bars). The mean initial viral population size is given by


(2) $$\bar{N}=\sum_{N=0}^{\infty }Np_{N}(\lambda ).$$

Similarly, we can plot the log-likelihood landscape as a function of the mean transmission bottleneck size $\bar{N_{b}}$ and *μ* (Fig. 3h). The expression for the mean transmission bottleneck size is provided in the Supplementary material. The mean transmission bottleneck size quantifies the mean number of initial viral particles that successfully establish genetic lineages in the recipient host.

Finally, we can use our maximum likelihood estimates of *λ* and *μ* to generate the predicted probability distribution for the number of clonal variants observed. We generate this estimated distribution using equation (1). Figure 3b shows this estimated distribution (light green bars) alongside the distribution from the simulated dataset (dark green bars). These distributions are quantitatively similar, indicating that our model, as parameterized, can recover the distribution of clonal variant outcomes observed in the mock dataset.

In the above analysis, we fixed $R_{0}$ at its true value of 1.6 and jointly estimated *μ* and *λ*. In a specific application to data, it might be the case that a literature estimate exists for *μ* but not the within-host basic reproduction number. We therefore assessed whether we could accurately infer within-host $R_{0}$ alongside *λ* while fixing *μ* at its true value of 0.4 mutations per genome per infection cycle. We found that our maximum likelihood estimates of $R_{0}$ and *λ* were again very close to their true values and that the true values of $R_{0}$ and *λ* again fell within our 95% confidence interval. However, the 95% confidence interval of *λ*, our primary parameter of interest, was considerably broader than when we set $R_{0}$ to its true value and jointly estimated *μ* and *λ* (Fig. 3i). Figure 3j and k, respectively, show these results as a function of $\bar{N}$ and $\bar{N_{b}}$ instead of *λ*.

Finally, we asked whether it would be possible to jointly estimate *λ*, *μ*, and $R_{0}$ based on the number of observed clonal variants across the mock dataset of transmission pairs. Supplementary figure S2A–C, Supplementary Material online shows the profile likelihoods for *λ*, $R_{0}$, and *μ*, respectively, over broad ranges. The profile likelihoods are remarkably flat, with the 95% confidence intervals on each of these parameters spanning across the shown ranges. These flat likelihood curves indicate that there are identifiability issues that arise when trying to jointly estimate all 3 of these parameters. To better understand why this is the case, we jointly estimated *μ* and *λ* while setting the within-host $R_{0}$ to different values: 1.3 (supplementary fig. S2D, Supplementary Material online), 1.6 (the true value; supplementary fig. S2E, Supplementary Material online), and 3 (supplementary fig. S2F, Supplementary Material online). While the maximum likelihood values across these 3 panels are very similar, the maximum likelihood estimates for (λ,μ) transition from a high *λ*, low *μ* combination at low $R_{0}$ to a low *λ*, high *μ* combination at high $R_{0}$. These results make sense in that a low $R_{0}$ results in a higher mean number of clonal variants (Fig. 2a and b). For a specific dataset, this leads to lower *μ* values and higher *λ* values, both of which tend to decrease the number of clonal variants observed. Analogously, a high $R_{0}$ results in a lower mean number of clonal variants (Fig. 2a and b). For a specific dataset, this leads to higher *μ* values and lower *λ* values, both of which tend to increase the number of clonal variants observed. Taken together, the results in supplementary fig. S2, Supplementary Material online therefore indicate that either the mutation rate or the within-host $R_{0}$ needs to be set to a reasonable value based on literature estimates to be able to make informative inferences about transmission bottleneck sizes using this approach.

### Application to Empirical Data

We apply our inference approach to 2 acutely infecting respiratory viruses: seasonal IAV and SARS-CoV-2. Application of our approach to an empirical dataset works analogously to the application of our approach to a simulated dataset. The only additional step involves the identification of clonal variants using virus deep-sequencing data from donor–recipient transmission pairs. Below, we use transmission pairs that have been previously identified using a combination of epidemiological, clinical, and viral genetic criteria. We identify clonal variants in these already-established transmission pairs by first setting a variant-calling threshold (e.g. 3%). We then call intrahost single nucleotide variants (iSNVs) for the donor sample and for the recipient sample based on this threshold. All sites that harbor an iSNV in either the donor or the recipient are then removed from consideration, as they are polymorphic in at least one of these individuals. At the remaining sites, we determine which allele is present in both the donor and the recipient. Any site that carries a different allele in the recipient compared to the donor is then called as a clonal variant.

### Application to IAV

**Fig. 4.**
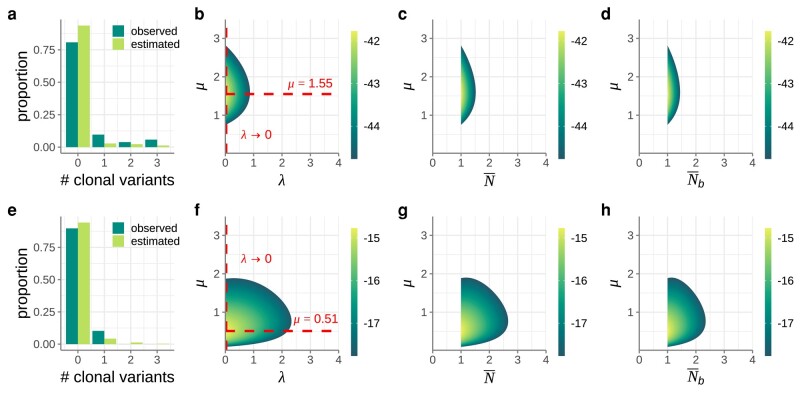
Application of our inference method to IAV and SARS-CoV-2 transmission pairs. Top row shows IAV results. Bottom row shows SARS-CoV-2 results. a) Distribution of the number of clonal variants observed across the 52 identified IAV transmission pairs (dark green bars). The expected distribution under the maximum likelihood estimates of *λ* and *μ* (light green bars) is shown alongside the empirical distribution. b) Log-likelihood plot, showing the log(probability) of observing the IAV dataset across a range of *λ* and *μ* values. Dashed red lines show the maximum likelihood values for *λ* and *μ*. The value of λ→0 was evaluated by assuming that 100% of successful transmissions started off with N=1 initial viral particles. c) Log-likelihood plot, as in panel b, with the results plotted as a function of $\bar{N}$ and *μ* instead of *λ* and *μ*. d) Log-likelihood plot, as in panel b, with the results plotted as a function of $\bar{N_{b}}$ and *μ* instead of *λ* and *μ*. The 95% confidence region of $\bar{N_{b}}$ spanned the range [1.00, 1.41]. e) Distribution of the number of clonal variants observed across the 39 identified SARS-CoV-2 transmission pairs (dark green bars). The expected distribution under the maximum likelihood estimates of *λ* and *μ* (light green bars) is shown alongside the empirical distribution. f) Log-likelihood plot, showing the log(probability) of observing the SARS-CoV-2 dataset across a range of *λ* and *μ* values. g) Log-likelihood plot, as in panel f, with the results plotted as a function of $\bar{N}$ and *μ* instead of *λ* and *μ*. h) Log-likelihood plot, as in panel f, with the results plotted as a function of $\bar{N_{b}}$ and *μ* instead of *λ* and *μ*. The 95% confidence region of $\bar{N_{b}}$ spanned the range [1.00, 2.31]. In panels b–d and f–h, only the log-likelihood values that fall within the 95% confidence region are shown.

As the first empirical application of our inference approach, we considered a rich IAV dataset from a prospective community-based cohort study (McCrone et al. 2018). The relevant portion of this dataset are 52 transmission pairs that were identified as part of this study (Supplementary material). For each of these transmission pairs, we calculated the number of clonal variants observed in the recipient using a variant-calling threshold of 3%. The data consisted of 42 transmission pairs with 0 clonal variants, 5 transmission pairs with 1 clonal variant, 2 transmission pairs with 2 clonal variants, 3 transmission pairs with 3 clonal variants, and 0 transmission pairs with 4 or more clonal variants (Fig. 4a). We set the within-host basic reproduction number $R_{0}$ to 11.1, based on a quantitative analysis of IAV dynamics in longitudinally studied human IAV infections (Baccam et al. 2006). We considered *λ* values in the range of (0, 4] mean initial viral particles and *μ* values between 0 and 3.5 mutations per genome per infection cycle. In practice, we considered a range in *λ* from 0.01 to 4 mean initial viral particles and then also evaluated the limit as *λ* approached 0 by calculating the likelihood with N=1 initial viral particles.

Figure 4b shows the log-likelihood surface for *λ* and *μ*. Figure 4c and d plots these same results as a function of $\bar{N}$ and $\bar{N_{b}}$, respectively. The log-likelihood surface shown in Fig. 4d corroborates previous results of very tight transmission bottlenecks for IAV (McCrone et al. 2018). Indeed, the maximum likelihood estimate of λ→0 indicates that almost all successful transmissions are predicted to have started with a single initial viral particle (N=1). Our results further provide an estimate of the mutation rate that is consistent with an independent mutation rate estimate obtained using a twelve class fluctuation test (Pauly et al. 2017). Specifically, the fluctuation test estimated the occurrence of 2 to 3 mutations on average per replicated genome. With approximately 30% of IAV mutations estimated to be lethal deleterious (Visher et al. 2016), we expect based on these results that *μ* be approximately (2−3)×0.70=1.4−2.1 mutations per replicated genome, consistent with our findings in Fig. 4b–d. Finally, we used our maximum likelihood estimates of *λ* and *μ* to generate the predicted probability distribution for the number of clonal variants observed. Figure 4a shows this predicted distribution alongside the distribution from the empirical IAV dataset. Although the predicted and empirical distributions are quantitatively similar, we note that our model, parameterized with maximum likelihood parameter values, appears to overestimate the proportion of clonal variants in the k=0 class and underestimate the proportion of clonal variants in higher-*k* classes.

Because our findings depend on our assumption of $R_{0}$, we reapplied our inference approach across a broader range of reasonable $R_{0}$ values. Supplementary figure S3, Supplementary Material online provides a sensitivity analysis of our *λ* and *μ* estimates under a range of $R_{0}=4.4$ to 37.7, corresponding to the minimum and maximum $R_{0}$ estimates in Baccam et al. (2006). This analysis indicates that our estimates are relatively insensitive to the exact value of $R_{0}$ assumed. Across the range of $R_{0}$ values considered, the maximum likelihood estimates of *λ* and associated mean transmission bottleneck sizes $\bar{N_{b}}$ remained low. The maximum likelihood estimates of the mutation rate also remained at similar values, with a slightly lower mutation rate estimated when $R_{0}$ was assumed to be low compared to when it was assumed to be high. Of note, the overestimation of the probability mass in the k=0 class (and the underestimation of the probability masses in the k≥1 classes) is less stark at lower $R_{0}$ values, indicating that literature estimates of within-host $R_{0}$ values may be high.

Because our sensitivity analyses indicate that the clonal variant data may support an $R_{0}$ value that is lower than current literature estimates, we decided to perform an additional analysis where we set the mutation rate and attempted to instead jointly estimate *λ* and the within-host basic reproduction number $R_{0}$ (supplementary fig. S4A–D, Supplementary Material online). With this analysis, we found that the maximum likelihood estimate for within-host IAV $R_{0}$ was 5.01, while the maximum likelihood estimate for *λ* remained at λ→0, corresponding to all successful transmissions starting off with a single initial viral particle (N=1). The maximum likelihood estimate of within-host $R_{0}=5.01$ is low compared to the range of individual estimates given in Baccam et al. (2006), although one of the 6 individuals studied in that analysis had an $R_{0}$ estimate lower than 5.01. As an additional consideration, the within-host $R_{0}$ we estimated in supplementary fig. S4, Supplementary Material online reflects the value of this parameter very early on during the infection process, while the within-host $R_{0}$ estimated in Baccam et al. (2006) is based on the exponential growth rate of the viral population once viral titers are sufficiently high to be detected with nasal washes. Due to changes in cellular multiplicities of infection over this period, and the effect this would have on viral complementation and competition, it might be the case that these within-host $R_{0}$ values are not immediately comparable.

### Application to SARS-CoV-2

Next, we applied our inference approach to a previously published SARS-CoV-2 transmission pair dataset from Austria (Popa et al. 2020). This dataset included 39 identified transmission pairs from early on in the SARS-CoV-2 pandemic (Supplementary material). Based on shared genetic variation between donors and recipients, transmission bottlenecks sizes were estimated to be tight (Martin and Koelle 2021; Nicholson et al. 2021), on the order of 1–3 viral particles. Here, we reanalyzed these same transmission pairs using our new inference approach, again using a variant-calling threshold of 3%. The data consisted of 35 transmission pairs with 0 clonal variants, 4 transmission pairs with 1 clonal variant, and 0 transmission pairs with 2 or more clonal variants (Fig. 4e). We set the within-host basic reproduction number $R_{0}$ to 7.4, based on a quantitative analysis of SARS-CoV-2 dynamics in longitudinally studied human SARS-CoV-2 infections (Ke et al. 2021). We again considered *λ* values in the range of (0, 4] initial viral particles and *μ* values between 0 and 3.5 mutations per genome per infection cycle.

Figure 4f shows the log-likelihood surface for *λ* and *μ*. Figure 4g and h plots these same results as a function of $\bar{N}$ and $\bar{N_{b}}$, respectively. The log-likelihood surface shown in Fig. 4h corroborates previous results of very tight transmission bottlenecks for SARS-CoV-2 (Braun et al. 2021b; Lythgoe et al. 2021; Martin and Koelle 2021; Nicholson et al. 2021; Bendall et al. 2023). It further provides an estimate of the mutation rate that is largely consistent with an independent mutation rate estimate of $1-5\times 10^{-6}$ per site per infection cycle (Amicone et al. 2022). This estimate translates to a mutation rate of approximately 0.03 to 0.15 mutations per genome per infection cycle. Again, with approximately 30% of these mutations likely being lethal deleterious, we expect *μ* to be approximately 0.02 to 0.10 mutations per infection cycle. While our maximum likelihood estimate of μ=0.52 exceeds this estimated range, our 95% confidence interval on *μ* extends into this range. Finally, we used our maximum likelihood estimates of *λ* and *μ* to again generate the predicted probability distribution for the number of clonal variants observed. Figure 4e shows this predicted distribution alongside the distribution from the empirical SARS-CoV-2 dataset. We again note that while the predicted and estimated distributions are quantitatively similar, our maximum likelihood parameter estimates appear to overestimate the proportion of clonal variants in the k=0 class, and underestimate the proportion of clonal variants in higher-*k* classes.

To determine the sensitivity of our findings to our assumption of $R_{0}=7.4$, we again reapplied our inference approach across a broader range of reasonable $R_{0}$ values. Supplementary figure S5, Supplementary Material online shows our results under a range of $R_{0}$ values that span 2.6 to 14.9, corresponding to the minimum and maximum $R_{0}$ estimates in Ke et al. (2021). Our results again indicate that our estimates are relatively insensitive to the exact value of $R_{0}$ assumed. As was the case with our IAV analysis, estimates of *μ* were slightly higher at higher within-host $R_{0}$ values. Again, the overestimation of the probability mass in the k=0 class (and the underestimation of the probability masses in the k≥1 classes) is reduced at lower $R_{0}$ values, again indicating that literature estimates of within-host $R_{0}$ values may be high.

As we did for the IAV dataset, we then again considered an alternative analysis where we set the mutation rate to an estimate from the literature and attempted to instead jointly estimate *λ* and the within-host basic reproduction number $R_{0}$ (supplementary fig. S4E–H, Supplementary Material online). With this analysis, we found that the maximum likelihood estimate for within-host SARS-CoV-2 $R_{0}$ was 1.21, while the maximum likelihood estimate for *λ* remained at λ→0.

### Guarding Against the Erroneous Calling of Clonal Variants

In our application to empirical data, our findings will depend not only on the assumed value of $R_{0}$ but also on the variant-calling threshold used. In particular, a lower variant-calling threshold has the potential to reduce the number of clonal variants called. This would occur if a site (in either the donor, the recipient, or both) goes from being called a clonal variant at a higher variant-calling threshold to being excluded from consideration at a lower variant-calling threshold because this former clonal variant is now instead called as an iSNV in one or both individuals. This brings us to the question of which variant-calling threshold should be used and how we can best guard against the erroneous calling of clonal variants when they might be transmitted from a donor instead of arising de novo in a recipient host. Here, we provide several approaches that can be used to address these concerns and to thereby assess the robustness of our conclusions.

The first approach is simply to perform a sensitivity analysis that uses different variant-calling thresholds to calculate the number of clonal variants in each of the identified transmission pairs. To provide an example of this approach, we recalled clonal variants for the IAV and SARS-CoV-2 datasets at variant-calling thresholds of 0.5% and 7% (supplementary figs. S6 and S7, Supplementary Material online). As anticipated, the number of clonal variants observed was lower at the 0.5% variant-calling threshold than at the 3% threshold and the number of clonal variants observed was higher at the 7% variant-calling threshold than at the 3% threshold. This was the case for both datasets. For the IAV dataset, neither the maximum likelihood estimate for *μ* nor the maximum likelihood estimate for *λ* was sensitive to the specific variant-calling threshold used. Furthermore, the 95% confidence interval did not change dramatically across the thresholds evaluated (supplementary fig. S6, Supplementary Material online). For the SARS-CoV-2 dataset, neither the maximum likelihood estimate for *μ* nor the maximum likelihood estimate for *λ* was sensitive to the specific variant-calling threshold used. The 95% confidence interval, however, did broaden at the lower variant-calling threshold of 0.5% (supplementary fig. S7, Supplementary Material online). Overall, this sensitivity analysis provides reassurance that our findings are largely robust to changes in the variant-calling threshold used.

The second approach is to examine the empirical frequencies of the clonal variants called at a given variant-calling threshold. If a clonal variant is present at 0% in a donor and present at 100% in a recipient, then it is very likely that it arose de novo within the recipient and spread to fixation within that individual (although we cannot exclude the possibility that this variant was present as an iSNV in the donor at the time of transmission but was present in 0% of donor reads at the time of sampling). If a clonal variant is present at 0% in a donor and close to 100% (but not fixed) in the recipient, it is likely that the clonal variant arose de novo in the recipient but had not quite yet fixed in the recipient by the time of sampling. In this case, we may want to keep on considering this variant as a clonal variant. Finally, if a clonal variant is observed at a frequency above 0% in the donor (regardless of whether it is fixed or close to fixation in the recipient), we may want to be more cautious about calling this variant a clonal variant. This is because the donor may have transmitted this iSNV to the recipient. Based on these considerations, we revisited both of the empirical datasets. In the IAV dataset, of the 18 clonal variants identified at the 3% variant-calling threshold, 12 were present at frequencies of 0% in the donor and 100% in the recipient. Five were present at frequencies of 0% in the donor and ≥99.1% in the recipient. The remaining variant was present at 2.3% frequency in the donor and fixed at 100% in the recipient. We therefore considered an additional analysis where we included only the 17 clonal variants that were present in the donor at 0% frequency. This analysis resulted in similar estimates of *μ* and *λ* as in our original analysis (supplementary fig. S8, Supplementary Material online). In the SARS-CoV-2 dataset, of the 4 clonal variants identified at the 3% variant-calling threshold, 3 were present at frequencies of 0% in the donor and ≥98.6% in the recipient. The remaining variant was present at 0.7% frequency in the donor and at 99.9% in the recipient. We therefore considered an additional analysis where we included only the 3 clonal variants that were present in the donor at 0% frequency. Again, this analysis resulted in similar estimates of *μ* and *λ* as in our original analysis (supplementary fig. S9, Supplementary Material online).

Finally, we would like to note that if an iSNV that was present at very low frequency in the donor was transmitted and observed as fixed in the recipient, this would itself point toward a very small transmission bottleneck. This is because the probability of a low-frequency iSNV being transmitted and fixing in the recipient is higher at low *N* than at high *N* (Supplementary material; supplementary fig. S10, Supplementary Material online). As such, even if a variant was erroneously called as a clonal variant when it was instead a low-frequency donor iSNV that was transmitted, its presence would consistently point toward a small transmission bottleneck size.

### Considering Alternative Distributions for the Initial Number of Viral Particles That Start an Infection

**Fig. 5.**
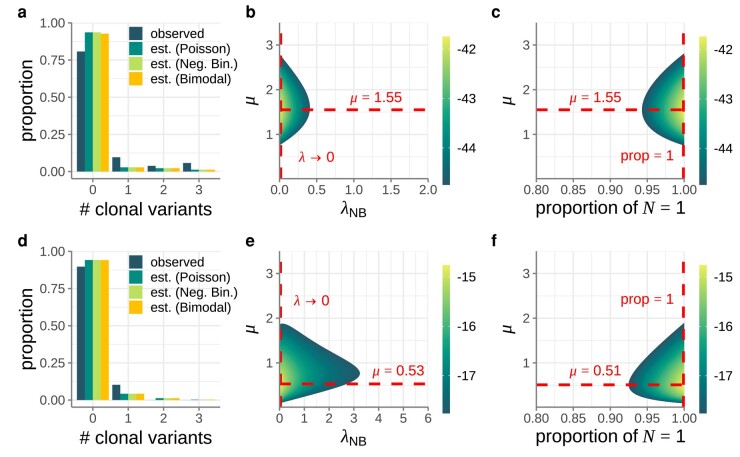
Consideration of alternative distributions for the number of initial viral particles *N*. Top row shows IAV results. Bottom row shows SARS-CoV-2 results. a) Distribution of the number of clonal variants observed across the 52 IAV transmission pairs considered (dark green bars). The expected distributions using the IAV maximum likelihood parameter estimates are shown in green, light green, and orange bars, respectively, for the Poisson distribution, negative binomial distribution, and bimodal (N=1 or *N*-large) distribution. b) Log-likelihood plot, showing the log(probability) of observing the IAV dataset across a range of $\lambda_{NB}$ and *μ* values. Dashed red lines show the maximum likelihood values for $\lambda_{NB}$ and *μ*. The parameter $\lambda_{NB}$ quantifies the mean of the negative binomial distribution. The overdispersion parameter of the negative binomial distribution was set to k=0.1. c) Log-likelihood plot, showing the log(probability) of observing the IAV dataset across a range of *p* and *μ* values. Dashed red lines show the maximum likelihood values for *p* and *μ*. The parameter *p* quantifies the proportion of recipients that start their infection off with N=1 initial viral particles. d) Distribution of the number of clonal variants observed across the 39 SARS-CoV-2 transmission pairs considered (dark green bars). The expected distributions using the SARS-CoV-2 maximum likelihood parameter estimates are shown in green, light green, and orange bars, respectively, for the Poisson distribution, negative binomial distribution, and bimodal distribution. e) Log-likelihood plot, showing the log(probability) of observing the SARS-CoV-2 dataset across a range of $\lambda_{NB}$ and *μ* values. Dashed red lines show the maximum likelihood values for $\lambda_{NB}$ and *μ*. The overdispersion parameter of the negative binomial distribution was set to k=0.1. f) Log-likelihood plot, showing the log(probability) of observing the SARS-CoV-2 dataset across a range of *p* and *μ* values. Dashed red lines show the maximum likelihood values for *p* and *μ*. For both datasets, a 3% variant-calling threshold was used to call clonal variants.

In our analysis of the mock dataset as well as in our analysis of the empirical IAV and SARS-CoV-2 datasets, we assumed that the initial number of viral particles *N* was Poisson-distributed. This assumption is consistent with an underlying process in which viral particles are transmitted from a donor at a constant rate during a contact event with fixed duration time. However, because of variation in the amount of virus a donor expels or because of differences in contact duration or transmission routes, a Poisson distribution for the number of initial viral particles might not be a good assumption. We therefore considered 2 alternative distributions for the initial number of viral particles. We first considered a highly overdispersed negative binomial distribution for *N*, with a large proportion of recipients receiving only very few initial viral particles and a small proportion receiving many initial viral particles, such that the variance of the distribution greatly exceeds its mean. Assuming this distribution, we attempted to jointly estimated its mean ($\lambda_{NB}$) and the mutation rate *μ* while setting the overdispersion parameter to a small value (k=0.1). For both the IAV and SARS-CoV-2 datasets, we found that the mean of this negative binomial distribution was low, similar to our findings using the Poisson distribution (Fig. 5b and e). We next considered a model in which a proportion *p* of recipients received N=1 viral particles from their donors, while the remaining proportion (1−p) received a large number of viral particles from their donors. We assumed that the number of initial viral particles in the latter recipients was so large that none of them would harbor a clonal variant. For both IAV and SARS-CoV-2 datasets, we found that the proportion of recipients receiving N=1 initial viral particles was very high (Fig. 5c and f). These additional analyses indicate that, while we do not know the underlying initial distribution of viral particles that recipients begin with, the pattern of clonal variants observed across transmission pairs points toward a large fraction of infected recipients starting their infection off with a very small number of initial viral particles. This does not exclude the possibility that a small fraction of infected recipients had their infections start off with a large number of initial viral particles, due to either contact with a donor that expelled a large amount of virus, a very long contact with a donor, or a contact of a specific type that could result in a large number of initial viral particles in the recipient.

## Discussion

Here, we developed a new statistical approach for estimating transmission bottleneck sizes from viral deep-sequencing data from donor–recipient transmission pairs. This approach differs from previous approaches in that it does not use the subset of viral sites that are identified as polymorphic in the donor. Instead, our approach relies on the number of clonal variants observed in the recipient. Observed clonal variants arise de novo shortly after transmission and are particularly well suited for estimating bottleneck sizes when bottlenecks are likely to be tight.

Our approach carries several advantages over existing approaches. First, transmission pairs where the donor does not show any genetic variation are still informative and can be included in our analysis. Second, a misspecification of donor versus recipient in a transmission pair does not impact results, as the number of clonal variants is the same with a correct donor/recipient assignment or the reverse. Third, existing studies that have looked at longitudinal viral samples have indicated that variant frequencies are highly dynamic over the course of an acute infection, consistent with a small within-host effective population size. As such, variant frequencies from a donor sample that are used to estimate bottleneck sizes may not reflect variant frequencies present in the donor at the time of transmission, and would lead to underestimates of $N_{b}$. Even if bottleneck sizes were large, changes in variant frequencies due to genetic drift in recipients would similarly bias $N_{b}$ estimates to be low. In contrast, our approach does not rely on variant frequencies in a donor, nor does it rely on variant frequencies in a recipient. As such, it is not subject to these same biases. Examination of our datasets also indicates that clonal variants remain clonal over the course of a recipient’s infection, such that the timing of the sampling event does not impact our dataset and thus does not impact our bottleneck size estimates. Finally, if viral particles from a donor are not randomly sampled, this does not impact our inference, while it would again bias $N_{b}$ estimates to be low with existing inference approaches.

Despite these advantages of our new inference approach, there are some limitations to it. First, estimation of transmission bottleneck sizes requires more than a single transmission pair. Second, our approach depends on a very limited subset of the donor and recipient deep-sequencing data. However, for the reasons we described above, we do not believe that patterns of shared genetic variation between the donor and the recipient are particularly informative of transmission bottleneck sizes, at least for acute viral respiratory infections such as influenza and SARS-CoV-2. Our approach does ignore de novo genetic variation that is subclonal in the recipient, however (that is, de novo variants that are called in a recipient but are not fixed). Our approach could, in principle, be extended to accommodate these variants. However, based on longitudinal analyses of IAV infections (McCrone et al. 2018), we also think that many of these subclonal variants come and go over the course of an infection, such that they are not informative of the transmission bottleneck size, but instead are more informative of the extent of genetic drift that occurs over the course of an acute infection. We thus do not recommend extension of our approach to accommodate subclonal variants. Additional limitations of our approach include our assumption of infinite sites and our assumption that all genetic variation is neutral. We do not believe that the infinite sites assumption would substantially bias our results because the number of clonal variants is very small compared to the length of the viral genome. We also do not believe that the neutrality assumption would substantially bias our results in the case of small transmission bottleneck sizes because genetic drift dominates in this regime, such that small fitness differences between viral particles will not impact the viral population’s evolutionary dynamics. Lethal deleterious mutations will simply act to lower the mutation rate estimate or decrease the effective within-host $R_{0}$ of the viral population.

Finally, our approach assumes complete genetic linkage across the viral genome. Again, we do not believe that this assumption would bias our results substantially, for several reasons. First, previous work has indicated that the effective rate of reassortment in human IAV infections is low even when viral titers are high (Sobel Leonard et al. 2017a), potentially due to spatial structure in the respiratory tract. As such, even if reassortment or recombination for viral respiratory pathogens is common, this genetic exchange likely occurs between genetically identical viral haplotypes and would therefore not bias our bottleneck size estimates that assume that linkage is complete. Second, if a mutation arises de novo in a recipient and fixes, this is very likely to occur within the first few viral replication cycles when the extent to which viral genetic diversification has occurred is limited and while viral population sizes in the recipient are still very small. Because of the small population sizes during this time period, the probability of high cellular multiplicity of infection may also be lower, and as such, the probability that recombination or reassortment occurs may be lower. If recombination or reassortment did occur early on during an infection, however, it could allow 2 mutations that arose de novo in different genetic backgrounds to come into the same background, allowing both to fix when in the absence of recombination/reassortment, neither would fix. By not considering this possibility, our estimate of $N_{b}$ would be biased low because we would ascribe the observation of 2 clonal variants in a recipient to be due to a small bottleneck rather than recombination/reassortment in the context of a larger bottleneck. Again, we think that the occurrence of this scenario is extremely rare, given that effective rates of recombination are likely to be low and that recombination/reassortment would have to occur very early on during infection when viral population sizes were still very small.

In our application to influenza A virus and to SARS-CoV-2, we found that transmission bottleneck sizes were very tight, consistent with previous findings of small $N_{b}$. This is an important finding, given that previous methods, as elaborated on above, are likely to underestimate bottleneck sizes due to within-host genetic drift and the potential for viral aggregation during the transmission process. That both this new approach and existing approaches arrive at the conclusion of very small bottleneck sizes does not lessen the advantage of using this new approach over existing approaches. If bottleneck sizes were large, and existing methods were considerably underestimating them, the IAV and SARS-CoV-2 datasets would not have contained any clonal variants. Our new approach would have indicated that for reasonable *μ* and within-host $R_{0}$ values, we would have anticipated observing clonal variants in a subset of the transmission pairs if bottleneck sizes were small. Their absence would therefore have argued against small bottleneck sizes and would have pointed toward previous methods indeed yielding biased estimates. That both sets of approaches (those based on shared genetic variation and the current one based on de novo clonal variants) infer very small transmission bottlenecks provides compelling evidence that these bottlenecks for acutely infecting respiratory viral pathogens are indeed incredibly small. This raises the question of what environmental and molecular mechanisms constrain transmission bottleneck sizes. Are the number of viral particles that reach the respiratory tract of a recipient limited? Or do many viral particles reach a recipient’s respiratory tract but host and/or viral factors limit the number of viral lineages that establish? Our results of tight transmission bottleneck sizes for IAV and SARS-CoV-2 also indicate that reductions in viral population sizes between transmission events will have a large impact on shaping these viruses’ patterns of evolution and adaptation at the population level. Will these small bottlenecks ultimately act to impede viral adaptation or to facilitate it? And how will these tight bottlenecks impact population-level viral patterns, including patterns of antigenic change, genetic diversification, and deleterious mutation loads? Addressing these questions through theoretical and empirical studies will facilitate our understanding of viral transmission dynamics and ultimately guide our ability to curb the spread of these infectious diseases.

## Supplementary Material

msad286_Supplementary_Data
