# Supplementary material for: Transmission Bottleneck Size Estimation from De Novo Viral Genetic Variation
Source: Mol Biol Evol. 2023 Dec 30;41(1):msad286. doi: 10.1093/molbev/msad286 (PMC10798134; doi:10.1093/molbev/msad286)
Supplement: msad286_Supplementary_Data [file msad286_supplementary_data.zip › Nb_from_clonal_denovo_supplemental.pdf]

Transmission bottleneck size estimation from *de novo* viral genetic variation - Supplemental Material

Yike Teresa Shi[1], Jeremy D. Harris[1,2], Michael A. Martin[1,3,4], Katia Koelle[1,5,*]

1 Department of Biology, Emory University, Atlanta, GA, USA
2 Department of Mathematics, Rose-Hulman Institute of Technology, Terre Haute, IN, USA
3 Department of Pathology, Johns Hopkins School of Medicine, Baltimore, MD, USA
4 Graduate Program in Population Biology, Ecology, and Evolution, Emory University, Atlanta, GA, USA
5 Emory Center of Excellence for Influenza Research and Response (CEIRR), Atlanta GA, USA

[*]katia.koelle@emory.edu

Derivation of the probability distribution for the number of clonal variants

Here, we derive the probabilities associated with the possible dynamic outcomes shown in Figure 1E and then show how these probabilities can then be used to obtain the probability distribution for the number of clonal variants. Consider first a contact between a donor and a recipient that results in the transfer of N infectious viral particles. Following this transfer, the within-host dynamics of the viral population depend on the within-host basic reproduction number R_0.

Because the initial viral population size may be small, we consider the viral population dynamics to be subject to demographic stochasticity. More specifically, we assume that the viral population undergoes stochastic birth-death dynamics. This is dynamically equivalent to a branching process model with a geometric offspring distribution parameterized with a success probability of $p_{geom} = 1/(R_0 + 1)$, where R_0 is the mean number of offspring produced (Lloyd-Smith *et al.*, 2005). When $R_0 < 1$, this corresponds to the subcritical case, and the viral population will die out with probability 1. In terms of the possible dynamic outcomes in Figure 1E, this corresponds to $P_X = 1$, regardless of the initial viral population size N. When $R_0 > 1$, this corresponds to the supercritical case. In this case, the viral population still has the possibility of going stochastically extinct. This occurs with probability:

$$P_{X,n=N} = (1/R_0)^N. \tag{1}$$

The probability of the viral population dying out P_X is the same as the probability of there being no mutant lineages that establish in the viral population (S_0), and corresponds to the scenario depicted in Figure 1A.

Because $P_X = 1$ in the subcritical case of $R_0 < 1$, we consider the remaining dynamic outcomes shown in Figure 1E only in the supercritical case. We can next derive the probability that the wild-type viral lineage successfully establishes (labeled S_∞), corresponding to the scenario depicted in Figure 1B. To calculate this probability, we have to consider the rate at which mutations occur during replication in the viral population. We assume a per genome, per infection cycle mutation rate μ, with the number of mutations occurring during the production of a viral progeny being Poisson-distributed with this mean. Given this assumption, the probability that a mutation does not occur during the production of a viral progeny is given by $e^{-\mu}$ and the probability that one or more mutations occur during the production of a viral progeny is given by $1 - e^{-\mu}$. Given this set-up, we can decompose the overall geometric offspring distribution into two separate offspring distributions: that of offspring that are genetically identical to the parent and that of offspring that differ from the parent by at least one mutation (Figure S1A). We define the wild-type offspring distribution as the distribution of offspring from wild-type individuals that are themselves wild-type. We further define the mutant offspring distribution as the distribution of offspring from wild-type individuals that instead carry *de novo* mutations. The wild-type offspring distribution is given by a negative binomial distribution with parameters $r_w = e^{-\mu}$ and success probability p_{geom}. The mutant offspring distribution is given by a negative binomial distribution with parameters $r_m = 1 - e^{-\mu}$ and success probability p_{geom}. If the number of wild-type offspring from a wild-type particle is $X \sim NB(r_w, p_{geom})$ and the number of mutant offspring from a wild-type particle is $Y \sim NB(r_m, p_{geom})$, the overall offspring distribution from a wild-type particle is $X + Y \sim NB(r_w + r_m, p_{geom}) = NB(1, p_{geom}) = Geom(p_{geom})$, thereby recovering the assumed overall offspring distribution.

Now that we have the wild-type offspring distribution defined, we can calculate the

probability that the wild-type viral lineage establishes. This is given by:

$$S_{\infty,n=N} = 1 - (p_{wtext,n=1})^N \tag{2}$$

where $p_{wtext,n=1}$ is the probability that a wild-type viral lineage, starting with a single infectious viral particle goes extinct. If the mean number of wild-type offspring (given by $R_0 e^{-\mu}$) exceeds one, this probability can be calculated numerically using equation (4) in Nishiura *et al.* (2012), using the parameters of the wild-type offspring distribution (r_w and p_{geom}). If the mean number of wild-type offspring is less than one, $p_{wtext,n=1} = 1$, and $S_{\infty,n=N} = 0$.

Now that we have derived $S_{\infty,n=N}$ and $S_{0,n=N}$, we turn to deriving $S_{1,n=N}$, $S_{2,n=N}$, $S_{3,n=N}$, etc. Deriving these probabilities is a bit more involved. Our general approach is to first calculate the final size distribution of wild-type particles, conditional on wild-type lineage extinction, and then to use this final size distribution to generate a probability mass function for the number of mutant lineages generated from the wild-type viral population. From this distribution, we calculate the probability mass function for the number of mutant lineages that establish. This probability mass function gives us $S_{0,n=N}$, $S_{1,n=N}$, $S_{2,n=N}$, etc., thus completing our analytical expressions for the possible dynamic outcomes shown in Figure 1E.

To first calculate the final size distribution of wild-type particles, we use previous analytical results derived in Nishiura *et al.* (2012) and Blumberg and Lloyd-Smith (2013b). For both the wild-type supercritical case ($R_0 e^{-\mu} > 1$) and the wild-type subcritical case ($R_0 e^{-\mu} < 1$), the final size distribution of the wild-type viral population, starting with one single infectious particle, is given by equation (1) in Blumberg and Lloyd-Smith (2013a) for a branching process model with a negative binomial offspring distribution (here, parameterized with r_w and p_{geom}). For the subcritical case, the probability masses of this distribution add up to 1. For the supercritical case, the probability masses of this distribution add up to $p_{wtext,n=1}$. We can thus normalize this latter distribution such that its probability masses add up to 1 (thereby conditioning on wild-type viral lineage extinction). We define this normalized probability distribution as $f_{n=1}$. Making use of $f_{n=1}$, we can then calculate the final size distribution of wild-type particles starting with N viral particles ($f_{n=N}$) through recursion:

$$f_{j,n=N} = \sum_{i=1}^{j-1} (f_{i,n=1} \times f_{j-i,n=N-1}) \tag{3}$$

with the terminal case being $f_{j,n=1}$. Here, $f_{j,n=N}$ refers to the probability mass of the final size of wild-type particles being j, given an initial viral population size of N. Figure S1B shows the probability mass function $f_{n=N}$.

Once we have the wild-type final size distribution $f_{n=N}$ (conditional on wild-type extinction), we can calculate the probability mass function for the number of mutant lineages that were *generated* directly from the wild-type population prior to its extinction. We can do this calculation using the mutant offspring distribution. Because the mutant offspring distribution is a negative binomial distribution with parameters r_m and p_{geom}, if the final size of the wild-type population was j, then the number of mutant offspring from this wild-type population itself follows a negative binomial distribution with parameters ($j \times r_m$) and p_{geom}. We can thus iterate over all possible wild-type final population sizes to calculate an overall distribution for the number of mutant viral lineages that are generated from this wild-type population that ultimately goes extinct. We define this overall distribution as $g_{n=N}$ (Figure S1C).

Finally, we can use $g_{n=N}$ to calculate the distribution of the number of mutant lineages that *establish*, which we refer to as $h_{n=N}$. Each mutant lineage has a probability of establishing of $1 - 1/R_0$, such that:

$$h_{j,n=N} = \sum_{m=0}^{\infty} [(g_{m,n=N}) \times Bin(j, m, 1 - 1/R_0)]. \tag{4}$$

for $j = 0, 1, 2, ...$ (Figure S1D). Here, $Bin(j, m, 1 - 1/R_0)$ is the binomial probability of observing j successes, given m trials and a probability of success of $1 - 1/R_0$. We can then calculate $S_{0,n=N}$, $S_{1,n=N}$, $S_{2,n=N}$, etc., from $h_{n=N}$ by:

$$S_{k,n=N} = (h_{k,n=N}) \times (1 - S_{\infty,n=N}) \tag{5}$$

where $k = 0, 1, 2, ...$ (but not ∞). This completes our analytical derivations for the outcomes depicted in Figure 1E.

As shown in Figure 1E, the probability of an infection going stochastically extinct in a recipient is given by:

$$P_{X,n=N} = S_{0,n=N} \tag{6}$$

The probability of there being zero clonal variants observed in a recipient is given by:

$$P_{0,n=N} = \sum_{i=2}^{\infty} S_{i,n=N} \tag{7}$$

The probability of there being 1 or more clonal variants is given by:

$$P_{1+,n=N} = S_{1,n=N} \tag{8}$$

These three probabilities are shown in Figure S1E. We now turn to resolving $P_{1+,n=N}$ into the probability of observing exactly $k = 1, 2, 3, ...$ clonal variants.

Given the mutation rate μ, the probability that exactly k mutations occurred during the production of the mutant lineage is given by:

$$\frac{Poiss(k, \mu)}{1 - Poiss(0, \mu)} \tag{9}$$

where $Poiss(k, \mu)$ is the Poisson probability of observing k mutations given a mutation rate of μ. To a first approximation, we can therefore write:

$$P_{k,n=N} \approx P_{1+,n=N} \times \left(\frac{Poiss(k, \mu)}{1 - Poiss(0, \mu)} \right) \tag{10}$$

for $k \geq 1$. This expression captures the possibility that more than one clonal variant arises during the generation of a mutant lineage from a wild-type particle. However, this expression is an approximation because there is a possibility that additional clonal variants arise following the generation of this mutant lineage (that is, during its establishment). We can correct for this possibility by modifying the above expression by probabilities of these additional clonal variants arising. Specifically, we can write the probability of there being *exactly* one clonal variant as the product of there being one mutation that occurs during the generation of the first mutant lineage and there being no additional clonal variants arising in this lineage that starts off with one mutant viral particle:

$$P_{1,n=N} = P_{1+,n=N} \times \left(\frac{Poiss(1, \mu)}{1 - Poiss(0, \mu)} \right) \times \left(\frac{P_{0,n=1}}{1 - P_{X,n=1}} \right) \tag{11}$$

We can similarly calculate the probability of there being *exactly* two clonal variants as the probability that there are exactly two mutations that arise during the generation of the first mutant lineage and no clonal variants that arise thereafter, plus the probability

that exactly one mutation arises during the generation of the first mutant lineage and exactly one clonal variant arising thereafter:

$$
\begin{aligned}
P_{2,n=N} = & P_{1+,n=N} \times \left(\frac{Poiss(2,\mu)}{1-Poiss(0,\mu)}\right) \times \left(\frac{P_{0,n=1}}{1-P_{X,n=1}}\right) + \\
& P_{1+,n=N} \times \left(\frac{Poiss(1,\mu)}{1-Poiss(0,\mu)}\right) \times \left(\frac{P_{1,n=1}}{1-P_{X,n=1}}\right)
\end{aligned} \tag{12}
$$

Luckily, we can directly calculate all of these terms, including $P_{1,n=1}$, which is given by:

$$
P_{1,n=1} = P_{1+,n=1} \times \left(\frac{Poiss(1,\mu)}{1-Poiss(0,\mu)}\right) \times \left(\frac{P_{0,n=1}}{1-P_{X,n=1}}\right) \tag{13}
$$

Next, the probability of observing *exactly* $k = 3$ clonal variants is given by the sum of the probability of 3 clonal variants arising during the generation of the first mutant lineage (and none thereafter), the probability of 2 clonal variants arising during the generation of the first mutant lineage (and 1 therefore), and the probability of 1 clonal variant arising during the generation of the first mutant lineage (and 2 thereafter). More generally, therefore, the probability of observing *exactly* k clonal variants is given by:

$$
P_{k,n=N} = (P_{1+,n=N}) \sum_{i=1}^{k} \left(\frac{Poiss(i,\mu)}{1-Poiss(0,\mu)}\right) \left(\frac{P_{k-i,n=1}}{1-P_{X,n=1}}\right) \tag{14}
$$

At this point, we now have $P_{X,n=N}$ and $P_{k,n=N}$, for $k \geq 0$. Because we would not observe infections in recipients in the case of $P_{X,n=N}$, the final probability mass distribution for the number of clonal variants observed in a recipient is given by:

$$
\rho_{k,n=N} = \left(\frac{P_{k,n=N}}{1-P_{X,n=N}}\right) \tag{15}
$$

for $k \geq 0$ (Figure S1F).

Code for this inference approach (in both R and Matlab) is available from our GitHub site: https://github.com/koellelab/nbclonal. This site also includes an R package to calculate clonal variant probabilities.

Rederivation of the Bozic *et al.* (2016) equation for the mean number of clonal variants

Bozic *et al.* (2016) derived an equation for the expected number of clonal variants under a scenario of a population size starting with a single individual ($N = 1$). In their work, the underlying model was a birth-death model with parameter δ defined as the ratio of death rate to birth rate (d/b) and parameter u defined as the probability of a mutation occurring during the production of an offspring. The equation they derived for the mean number of clonal variants is given by their equation (46): $\delta u/(1-\delta)$.

Our underlying model is a branching process model, parameterized with a geometric offspring distribution. This distribution corresponds to the offspring distribution realized in a birth-death model. Our branching process model has parameter R_0 defined as the ratio of birth rate to death rate (such that $1/R_0$ is the ratio of death rate to birth rate, δ). Our model also has parameter μ, defined as the mean number of mutations that occur at birth, with the distribution of mutations occurring at birth being Poisson distributed. The probability that zero mutations occur during the production of an offspring is thus given as $e^{-\mu}$. When μ is small, the probability that a (single) mutation occurs during the production of an offspring is $1 - e^{-\mu}$, which is approximately μ. As such, when μ is small, it is approximately equal to Bozic et al.'s u parameter. Finally, to

correspond with the assumption in (Bozic *et al.*, 2016) of the population starting with a single individual, we set the initial viral population size N to 1.

With a small mutation rate μ, the possible outcomes shown in Figure 1E consist primarily of S_∞, S_0, and S_1, and the probability that more than one clonal variant establishes within the S_1 outcome is negligibly small. As such, the mean number of clonal variants is given by S_1 conditional on infection, such that $\bar{m}_{clonal} = S_1/(1 - S_0)$. This is equivalent to $\bar{m}_{clonal} = (1 - S_\infty - S_0)/(1 - S_0)$. As detailed above, $S_0 = 1/R_0$ when $N = 1$. When μ is small, we can approximate S_∞ as $1 - 1/(R_0 e^{-\mu}) = 1 - 1/(R_0(1-\mu))$. Substituting, the numerator becomes: $(1 - S_\infty - S_0) = \mu/R_0 = \delta u$, and the denominator becomes $(1 - S_0) = 1 - 1/R_0 = 1 - \delta$. As such, the expected number of clonal variants from our analytical expressions, parameterized with $N = 1$, agree with equation (46) provided in Bozic *et al.* (2016).

Calculation of the mean transmission bottleneck size $\bar{N}_b$

Then mean bottleneck size is given by:

$$\bar{N}_b = \sum_{b=1}^{\infty} b p_b(\lambda, R_0) \tag{16}$$

where $p_b(\lambda, R_0)$ denotes the probability that the bottleneck size is b in a successful infection. In turn, $p_b(\lambda, R_0)$ is given by:

$$p_b(\lambda, R_0) = \sum_{N=b}^{\infty} p_N(\lambda) Pr(b, N, 1 - 1/R_0)/[1 - Pr(0, N, 1 - 1/R_0)] \tag{17}$$

where $Pr(b, N, 1 - 1/R_0)$ is given by the binomial probability that exactly b out of the N initial viral particles successfully leave genetic lineages in the recipient host. This binomial probability is calculated using a success probability of $1 - 1/R_0$ for each initial viral particle.

Quantification of the number of clonal variants for the influenza A virus data set

We applied our inference approach to a previously published IAV transmission pair data set from Michigan, USA (McCrone *et al.*, 2018). The raw sequencing data have previously been made available by the authors (SRA BioProject: PRJNA412631). Individuals in the same household were considered transmission pairs if they displayed symptoms within 7 days of one another, were both positive for the same IAV subtype, and were infected with viruses that were genetically more similar to one another than 95% of epidemiologically unlinked pairs based on the L1-norm. Transmission pairs with multiple putative donors for a single recipient were discarded. Further, pairs in which both individuals had symptom onset on the same day were discarded unless those were the first two positive individuals in their household. In these cases (N=6), both possible orderings of donor and recipient were evaluated. In total, our analysis included 46 unique transmission pairs and 52 possible orderings of donor-recipient transmissions.

Table S1 provides a list of the clonal variants identified in these transmission pairs and the frequencies that these clonal variants were observed at prior to the variant-calling threshold being applied. We used the datafile no_cut_trans_freq.csv from McCrone *et al.* (2018)'s GitHub to calculate the number of clonal variants provided in this supplemental table.

Quantification of the number of clonal variants for the SARS-CoV-2 data set

We applied our inference approach to a previously published SARS-CoV-2 transmission pair data set from Austria (Popa *et al.*, 2020). The raw sequencing data have previously been made available by the authors (SRA BioProject: PRJEB39849). To establish transmission pairs, the authors combined information on intrafamily cases with information on epidemiological transmission chains. Additional telephone investigations were used to validate inferred transmission pairs. The data set consisted of 39 transmission pairs as reported in Data File S4 from (Popa *et al.*, 2020). Sequencing reads were downloaded from the SRA and variants were called relative to Wuhan/Hu-1 (NC_045512.2) as described in (Martin and Koelle, 2021). Table S2 provides a list of the clonal variants identified in these 39 transmission pairs and the frequencies that these clonal variants were observed at prior to the variant-calling threshold being applied. (Note that we have previously analyzed this data set using a 6% variant-calling threshold (Martin and Koelle, 2021). The reason we used this high threshold previously was to remove spurious low frequency variants that were shared across many of the samples and therefore inflated transmission bottleneck size estimates based on shared genetic variation. Here, we use the variant-calling threshold to identify the sites that are monomorphic in both the donor and the recipient and therefore might be sites that harbor clonal variants in the recipient. As such, a lower variant-calling threshold yields a more conservative estimate on the number of clonal variants observed. In contrast, a higher variant-calling threshold yields a more conservative estimate of shared genetic variation.)

Probability of a donor iSNV transmitting and fixing in a recipient

We can calculate the probability that an iSNV that is observed at low frequency in the donor transmits and fixes in the recipient. This probability depends on the frequency of the iSNV in the donor (q), the initial number of viral particles N and the within-host basic reproduction number R_0. The probability is given by:

$$\frac{\sum_{k=1}^{N} \left[Binom(k, N, q)(1/R_0)^{N-k}(1 - Binom(0, k, 1 - 1/R_0))\right]}{1 - P_X} \tag{18}$$

Figure S10 shows this probability over a range of N for IAV ($R_0 = 11.1$) and for SARS-CoV-2 ($R_0 = 7.4$). In both cases, it is clear that the probability that a low-frequency iSNV transmits and fixes is higher at lower N. This indicates that, if present, these iSNVs (which have the potential to be misidentified as clonal variants) are themselves evidence of small transmission bottleneck sizes. Note that the probability expression above assumes random sampling of viral particles from the donor.

References

Amicone, M., Borges, V., Alves, M. J., Isidro, J., Zé-Zé, L., Duarte, S., Vieira, L., Guiomar, R., Gomes, J. P., and Gordo, I. 2022. Mutation rate of SARS-CoV-2 and emergence of mutators during experimental evolution. *Evolution, Medicine, and Public Health*, 10(1): 142–155.

Blumberg, S. and Lloyd-Smith, J. O. 2013a. Comparing methods for estimating r_0 from the size distribution of subcritical transmission chains. *Epidemics*, 5(3): 131–145.

Blumberg, S. and Lloyd-Smith, J. O. 2013b. Inference of r_0 and transmission heterogeneity from the size distribution of stuttering chains. *PLoS Computational Biology*, 9(5): e1002993.

Bozic, I., Gerold, J. M., and Nowak, M. A. 2016. Quantifying clonal and subclonal passenger mutations in cancer evolution. *PLoS Computational Biology*, 12(2): e1004731.

Lloyd-Smith, J. O., Schreiber, S. J., Kopp, P. E., and Getz, W. M. 2005. Superspreading and the effect of individual variation on disease emergence. *Nature*, 438(7066): 355–359.

Martin, M. A. and Koelle, K. 2021. Comment on "genomic epidemiology of superspreading events in austria reveals mutational dynamics and transmission properties of SARS-CoV-2". *Science Translational Medicine*, 13(617): eabh1803.

McCrone, J. T., Woods, R. J., Martin, E. T., Malosh, R. E., Monto, A. S., and Lauring, A. S. 2018. Stochastic processes constrain the within and between host evolution of influenza virus. *eLife*, 7: e35962.

Nishiura, H., Yan, P., Sleeman, C. K., and Mode, C. J. 2012. Estimating the transmission potential of supercritical processes based on the final size distribution of minor outbreaks. *Journal of Theoretical Biology*, 294: 48–55.

Pauly, M. D., Procario, M. C., and Lauring, A. S. 2017. A novel twelve class fluctuation test reveals higher than expected mutation rates for influenza A viruses. *eLife*, 6: e26437.

Popa, A., Genger, J. W., Nicholson, M. D., Penz, T., Schmid, D., Aberle, S. W., Agerer, B., Lercher, A., Endler, L., Colaço, H., Smyth, M., Schuster, M., Grau, M. L., Martínez-Jiménez, F., Pich, O., Borena, W., Pawelka, E., Keszei, Z., Senekowitsch, M., Laine, J., Aberle, J. H., Redlberger-Fritz, M., Karolyi, M., Zoufaly, A., Maritschnik, S., Borkovec, M., Hufnagl, P., Nairz, M., Weiss, G., Wolfinger, M. T., von Laer, D., Superti-Furga, G., Lopez-Bigas, N., Puchhammer-Stöckl, E., Allerberger, F., Michor, F., Bock, C., and Bergthaler, A. 2020. Genomic epidemiology of superspreading events in austria reveals mutational dynamics and transmission properties of SARS-CoV-2. *Science Translational Medicine*, 12(573): eabe2555.

Supplemental Tables

Table S1. Table of IAV clonal variants identified in the set of transmission pairs from McCrone *et al.* (2018). For each clonal variant, the transmission pair in which it was identified is provided as are the frequencies of the variant in the donor and in the recipient.

Table S2. Table of SARS-CoV-2 clonal variants identified in the set of transmission pairs from Popa *et al.* (2020). For each clonal variant, the transmission pair in which it was identified is provided as are the frequencies of the variant in the donor and in the recipient.

Supplemental Figures

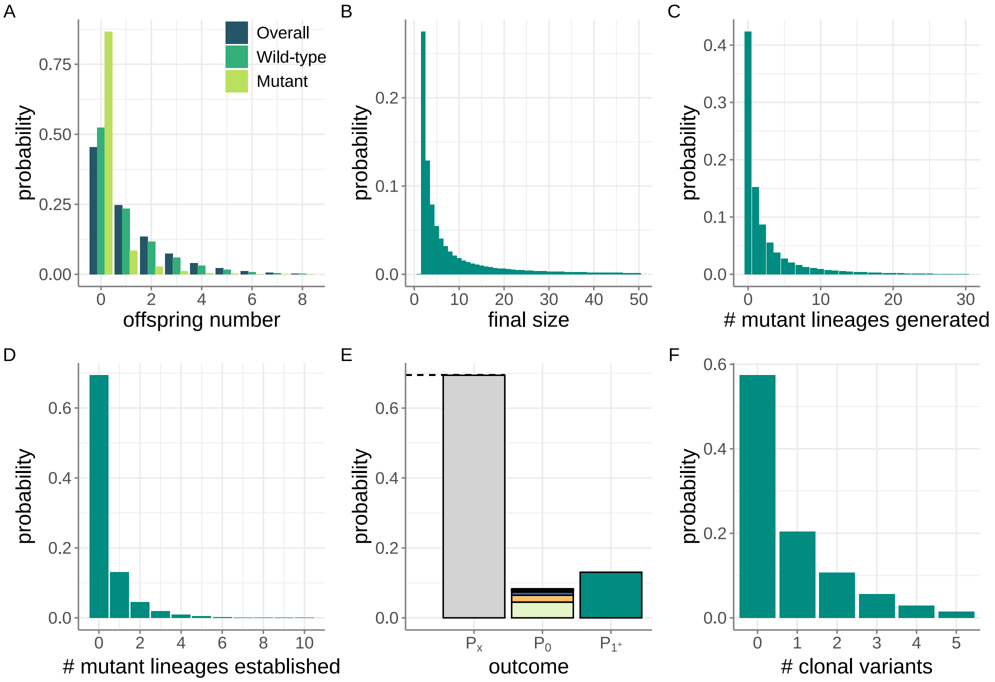

Figure S1. Steps involved in the derivation of the probability mass function for the number of clonal variants. (A) The overall offspring distribution, shown alongside the wild-type offspring distribution and the mutant offspring distribution. Here, the overall offspring distribution is a geometric distribution with mean $R_0 = 1.2$ (such that p_{geom} =0.4545). The wild-type offspring distribution and the mutant offspring distribution are both negative binomial distribution with r_w and r_m calculated using a mutation rate of $\mu = 0.2$. (B) The final size distribution of wild-type particles, conditional on wild-type lineage extinction. Here, the initial number of viral particles $N = 2$. (C) The probability mass function for the number of mutant lineages generated by the wild-type viral population, conditional on wild-type lineage extinction. (D) The probability mass function for the number of mutant lineages that successfully establish, conditional on wild-type lineage extinction. (E) Calculated probabilities of the overall viral population going extinct (P_X, as calculated by S_0), of the viral population establishing with zero clonal variants (P_0), and of the viral population establishing with one or more clonal variants (P_{1+}). Dashed black line shows the analytical calculation of P_X via the expression given by equation (3), indicating agreement with the calculated value of S_0. (F) Probability mass function for the number of clonal variants that establish in a recipient who becomes successfully infected.

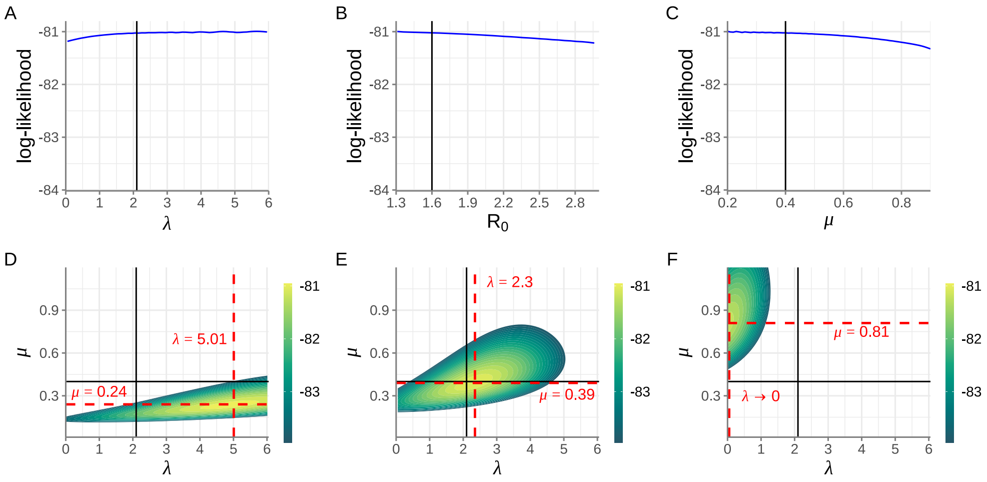

Figure S2. Attempt at joint estimation of λ, R_0, and μ. (A) Profile likelihood plot, showing the log(probability) of observing the mock data set across a range of λ values. (B) Profile likelihood plot, showing the log(probability) of observing the mock data set across a range of R_0 values. (C) Profile likelihood plot, showing the log(probability) of observing the mock data set across a range of μ values. In A-C, black vertical lines show the true parameter values used in the generation of the mock data set. (D) Log-likelihood plot, showing the log(probability) of observing the mock data set given parameters λ and μ. Here, R_0 was (erroneously) set to 1.3. (E) Log-likelihood plot, showing the log(probability) of observing the mock data set given parameters λ and μ. Here, R_0 was set to its true value of 1.6. (E) Log-likelihood plot, showing the log(probability) of observing the mock data set given parameters λ and μ. Here, R_0 was (erroneously) set to 3.0. In D-F, black lines show the true values of λ and μ and red dashed lines show the maximum likelihood estimates of λ and μ.

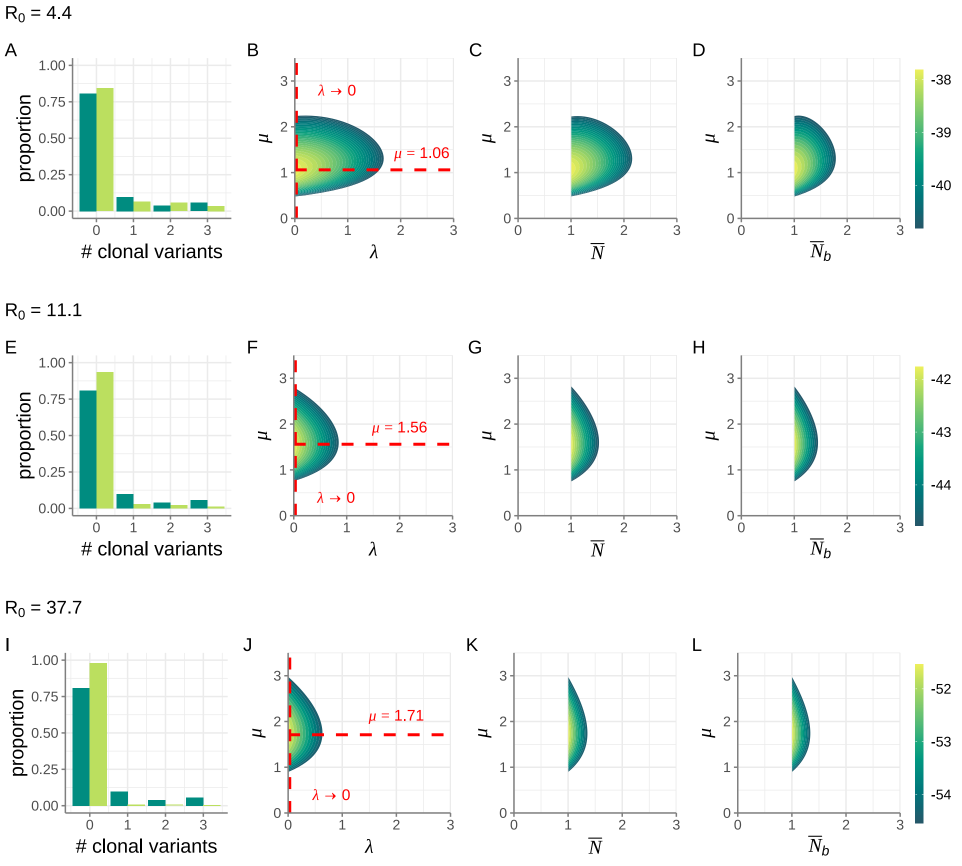

Figure S3. Sensitivity analysis of IAV results across different assumptions of within-host R_0. Results are shown in three rows, with the first row showing results with an assumed R_0 value of 4.4, the second row showing results with an assumed R_0 value of 11.1, and the third row showing results with an assumed R_0 value of 37.7. For each R_0 value considered, we plot four panels, analogous to those shown in the top row of Figure 4.

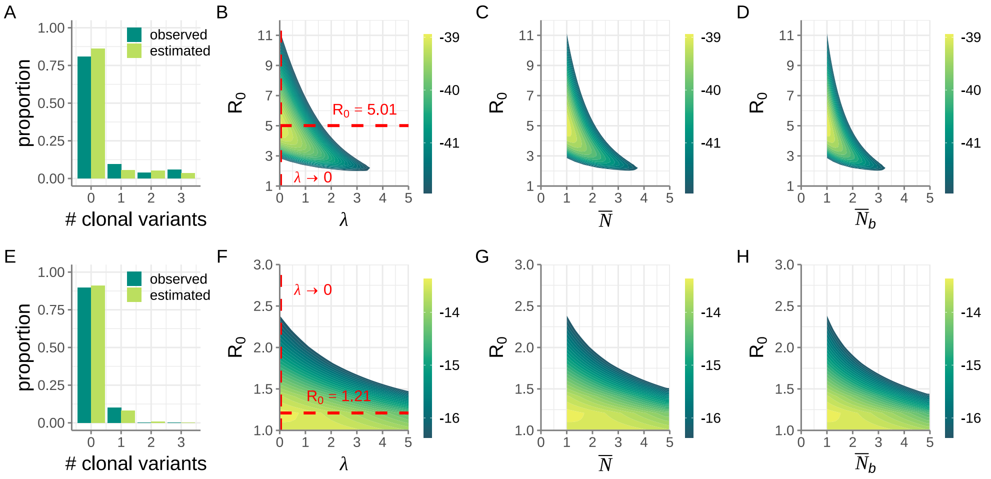

Figure S4. Application of our inference method to influenza A virus (top row) and SARS-CoV-2 (bottom row) transmission pairs. Here, we estimate λ and the within-host R_0, while setting the mutation rate μ to estimates from the literature. (A) Distribution of the number of clonal variants observed across the 52 identified IAV transmission pairs (dark green bars). The expected distribution under the maximum likelihood estimates of λ and R_0 (light green bars) is shown alongside the empirical distribution. (B) Log-likelihood plot, showing the log(probability) of observing the IAV data set across a range of λ and within-host R_0 values. Dashed red lines show the maximum likelihood values for λ and R_0. (C) Log-likelihood plot, as in panel B, with the results plotted as a function of $\bar{N}$ and R_0 instead of λ and R_0. (D) Log-likelihood plot, as in panel B, with the results plotted as a function of $\bar{N}_b$ and R_0 instead of λ and R_0. (E) Distribution of the number of clonal variants observed across the 39 identified SARS-CoV-2 transmission pairs (dark green bars). The expected distribution under the maximum likelihood estimates of λ and within-host R_0 (light green bars) is shown alongside the empirical distribution. (F) Log-likelihood plot, showing the log(probability) of observing the SARS-CoV-2 dataset across a range of λ and R_0 values. (G) Log-likelihood plot, as in panel F, with the results plotted as a function of $\bar{N}$ and R_0 instead of λ and R_0. (H) Log-likelihood plot, as in panel F, with the results plotted as a function of $\bar{N}_b$ and R_0 instead of λ and R_0. In panels B-D and F-H, only the log-likelihood values that fall within the 95% confidence region are shown. For inference on the IAV data set, we set the mutation rate to $\mu = 1.75$ mutations per genome per infection cycle, based on estimates from Pauly *et al.* (2017). For inference on the SARS-CoV-2 dataset, we set the mutation rate to $\mu = 0.03$ mutations per genome per infection cycle, based on estimates from Amicone *et al.* (2022).

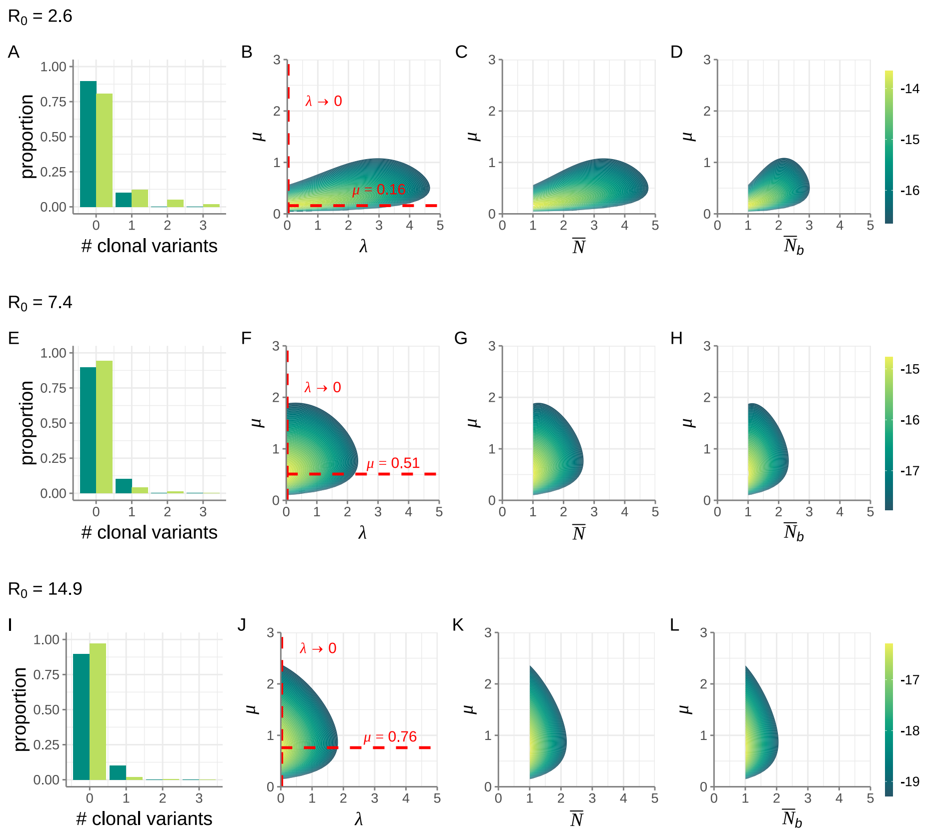

Figure S5. Sensitivity analysis of SARS-CoV-2 results across different assumptions of within-host R_0. Results are shown in three rows, with the first row showing results with an assumed R_0 value of 2.6, the second row showing results with an assumed R_0 value of 7.4, and the third row showing results with an assumed R_0 value of 14.9. For each R_0 value considered, we plot four panels, analogous to those shown in the bottom row of Figure 4.

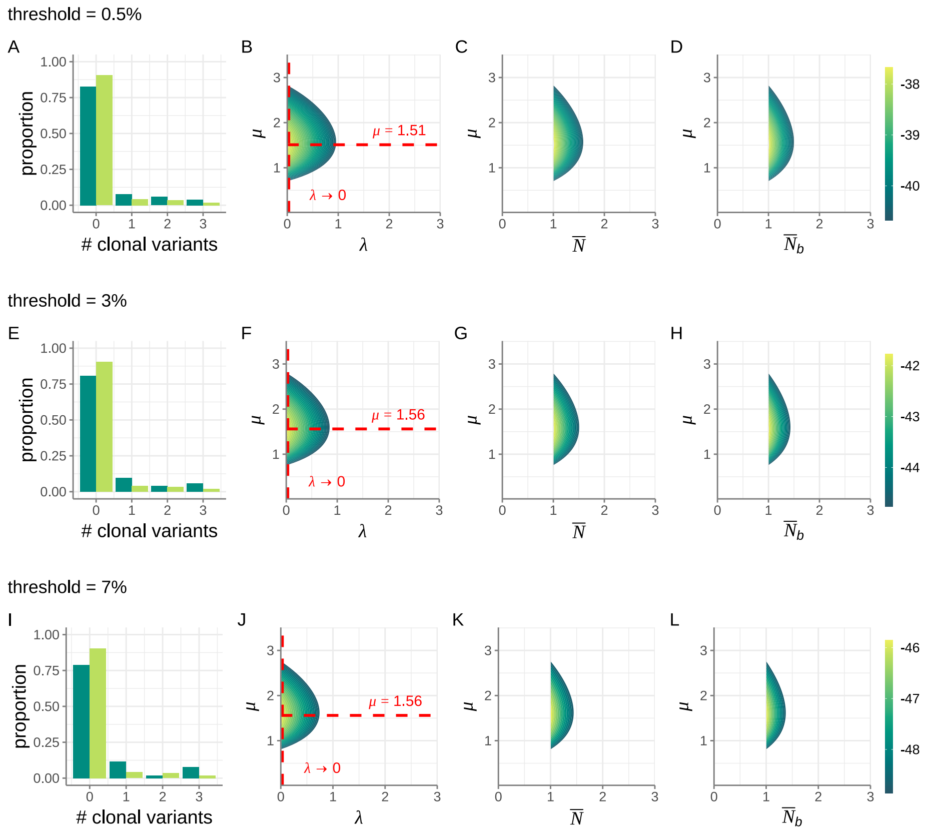

Figure S6. Sensitivity analysis of IAV results across different variant-calling thresholds used. (A-D) Inference results, as in Figures 4A-D, applying a variant calling threshold of 0.5%. (E-H) Inference results, as in Figures 4A-D, applying a variant calling threshold of 3%. (Identical to Figures 4A-D.) (I-L) Inference results, as in Figures 4A-D, applying a variant calling threshold of 7%. Notice that the distribution of the number of clonal variants observed across the transmission pairs can and does change with a change in the variant calling threshold applied.

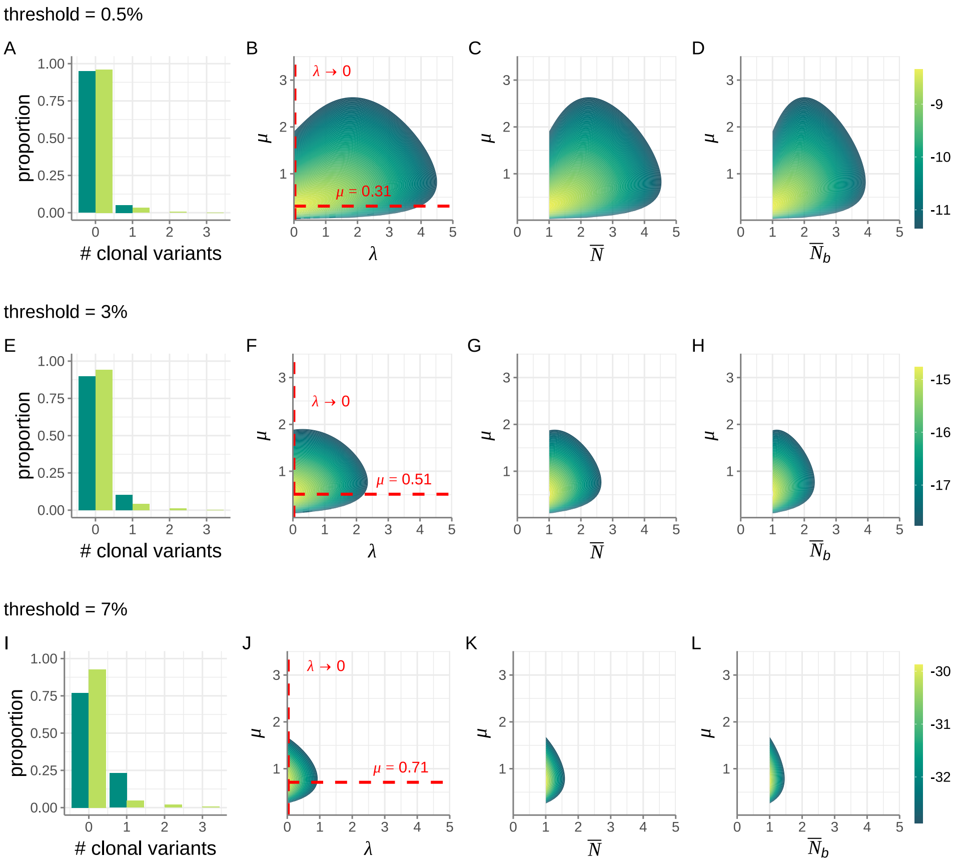

Figure S7. Sensitivity analysis of SARS-CoV-2 results across different variant-calling thresholds used. (A-D) Inference results, as in Figures 4E-H, applying a variant calling threshold of 0.5%. (E-H) Inference results, as in Figures 4E-H, applying a variant calling threshold of 3%. (Identical to Figures 4E-H.) (I-L) Inference results, as in Figures 4E-H, applying a variant calling threshold of 7%. Notice that the distribution of the number of clonal variants observed across the transmission pairs can and does change with a change in the variant calling threshold applied.

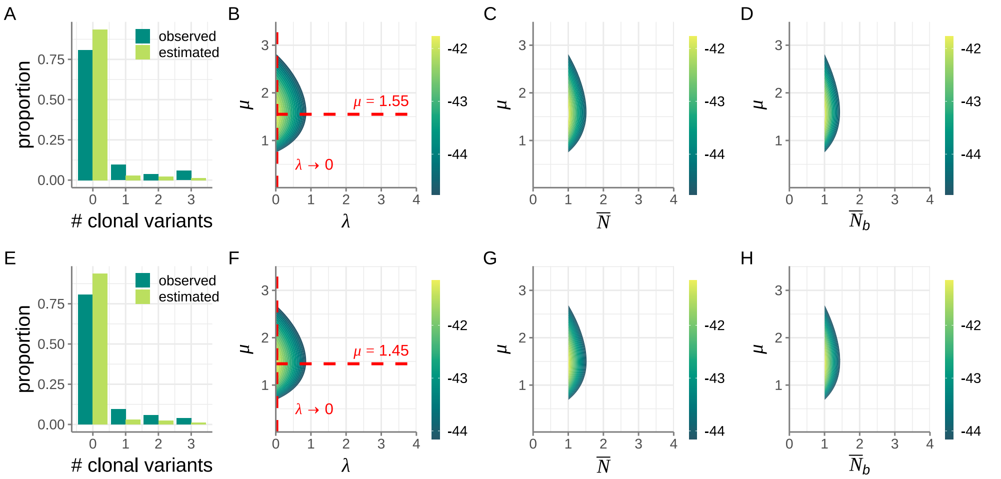

Figure S8. Sensitivity analysis of IAV results when removing the potentially spurious clonal variant that is present in the donor at a frequency of 2.3%. Top row reproduces the results shown in Figure 4 A-D. Bottom row shows inference results when this potentially spurious clonal variant was removed from the data set. Removal of this clonal variant resulted in a transmission pair previously classified as harboring three clonal variants being reclassified as harboring two clonal variants.

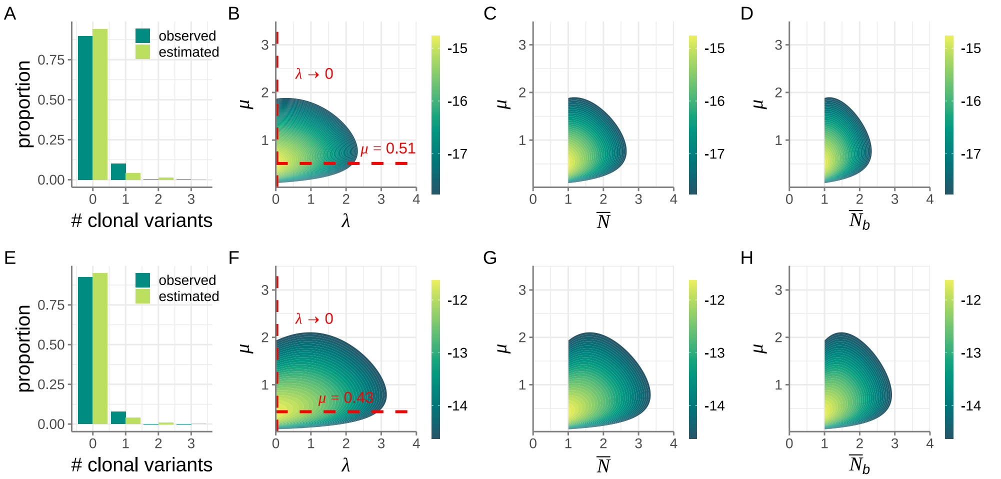

Figure S9. Sensitivity analysis of SARS-CoV-2 results when removing the potentially spurious clonal variant that is present in the donor at a frequency of 0.7%. Top row reproduces the results shown in Figure 4 E-H. Bottom row shows inference results when this potentially spurious clonal variant was removed from the data set. Removal of this clonal variant resulted in a transmission pair previously classified as harboring one clonal variant being reclassified as harboring no clonal variants.

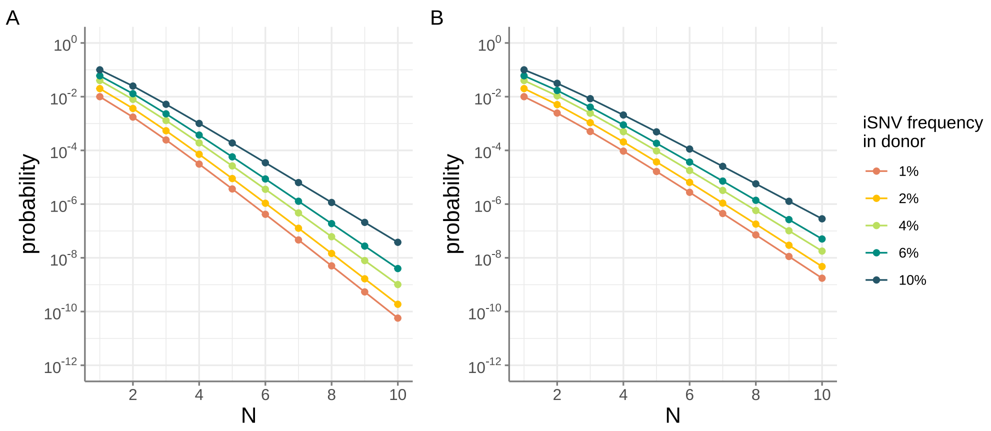

Figure S10. Probability that a donor-derived iSNV transmits to the recipient and fixes, as a function of the initial number of viral particles that start off the infection in the donor. (A) Probabilities that an IAV iSNV present in a donor at 1%, 2%, 4%, 6%, and 10% transmits to the recipient and fixes. (B) Probabilities that a SARS-CoV-2 iSNV present in a donor at 1%, 2%, 4%, 6%, and 10% transmits to the recipient and fixes. In (A), the within-host R_0 is set to 11.1. In (B), the within-host R_0 is set to 7.4.
